# Hyperexcitability and Homeostasis in Fragile X Syndrome

**DOI:** 10.3389/fnmol.2021.805929

**Published:** 2022-01-06

**Authors:** Xiaopeng Liu, Vipendra Kumar, Nien-Pei Tsai, Benjamin D. Auerbach

**Affiliations:** ^1^Deparment of Molecular & Integrative Physiology, University of Illinois at Urbana-Champaign, Urbana, IL, United States; ^2^Beckman Institute for Advanced Science & Technology, University of Illinois at Urbana-Champaign, Urbana, IL, United States

**Keywords:** fragile X syndrome, circuit hyperexcitability, homeostatic plasticity, E/I balance, sensory hypersensitivity, epilepsy

## Abstract

Fragile X Syndrome (FXS) is a leading inherited cause of autism and intellectual disability, resulting from a mutation in the *FMR1* gene and subsequent loss of its protein product FMRP. Despite this simple genetic origin, FXS is a phenotypically complex disorder with a range of physical and neurocognitive disruptions. While numerous molecular and cellular pathways are affected by FMRP loss, there is growing evidence that circuit hyperexcitability may be a common convergence point that can account for many of the wide-ranging phenotypes seen in FXS. The mechanisms for hyperexcitability in FXS include alterations to excitatory synaptic function and connectivity, reduced inhibitory neuron activity, as well as changes to ion channel expression and conductance. However, understanding the impact of *FMR1* mutation on circuit function is complicated by the inherent plasticity in neural circuits, which display an array of homeostatic mechanisms to maintain activity near set levels. FMRP is also an important regulator of activity-dependent plasticity in the brain, meaning that dysregulated plasticity can be both a cause and consequence of hyperexcitable networks in FXS. This makes it difficult to separate the direct effects of *FMR1* mutation from the myriad and pleiotropic compensatory changes associated with it, both of which are likely to contribute to FXS pathophysiology. Here we will: (1) review evidence for hyperexcitability and homeostatic plasticity phenotypes in FXS models, focusing on similarities/differences across brain regions, cell-types, and developmental time points; (2) examine how excitability and plasticity disruptions interact with each other to ultimately contribute to circuit dysfunction in FXS; and (3) discuss how these synaptic and circuit deficits contribute to disease-relevant behavioral phenotypes like epilepsy and sensory hypersensitivity. Through this discussion of where the current field stands, we aim to introduce perspectives moving forward in FXS research.

## Introduction

Fragile X syndrome (FXS) is the most common inherited form of intellectual disability (ID) and one of the leading known genetic causes of autism spectrum disorders (ASD; Hagerman et al., 2017). FXS is most commonly caused by the expansion and hyper-methylation of CGG-repeats around the *FMR1* gene, leading to its transcriptional silencing and the subsequent loss of its protein product, Fragile x mental retardation protein (FMRP; Bhakar et al., 2012). In rare cases, FXS can also arise from point mutations or deletions in the *FMR1* gene (Hammond et al., 1997; Myrick et al., 2014, 2015; Suhl and Warren, 2015). FMRP is a well-conserved neuronal RNA-binding protein involved in the transport and translational regulation of a large number of mRNA in the brain (Ashley et al., 1993; Siomi et al., 1993; Stefani et al., 2004; Santoro et al., 2012). The known genetics of FXS and the evolutionarily conserved nature of FMRP have allowed for the development of well-validated animals models of the disorder (Bhogal and Jongens, 2010; Schroeder et al., 2017). FXS has thus emerged as a prototype for a molecular medicine approach to neuropsychiatric disorders, i.e., treating diseases with complex pathophysiology by targeting underlying molecular and cellular alterations identified in pre-clinical models (Krueger and Bear, 2011). However, recent clinical trial failures in FXS have also underscored the potential pitfalls of attempting to translate therapies developed from molecular pathology identified in animal models into suitable clinical treatments (Berry-Kravis et al., 2018). These setbacks highlight the need for further understanding of how cellular and molecular perturbations caused by loss of FMRP contribute to neural circuit dysfunction in FXS, as these circuit abnormalities are most relevant to understanding how the behavioral phenotypes associated with FXS arise. Elucidating the consequences of *FMR1* mutation at the circuit and behavioral level is complicated by the wide-ranging, multifunctional role of FMRP as well as the vast compensatory mechanisms utilized by the brain to maintain neuronal function within an optimal range.

FMRP is highly enriched in neurons and expressed across various cell compartments, cell-types, and brain regions (Abitbol et al., 1993; Devys et al., 1993; Verheij et al., 1995; Feng et al., 1997; Christie et al., 2009; Olmos-Serrano et al., 2010). FMRP expression is also developmentally regulated in both humans (Abitbol et al., 1993) and mice (Saffary and Xie, 2011), with expression starting at early embryonic stages, peaking during early post-natal developmental critical periods, but remaining at sustained levels throughout adulthood (Till, 2010; Bonaccorso et al., 2015; Gholizadeh et al., 2015). Most evidence indicates that FMRP is a translation repressor, with the ability to inhibit both translation initiation (Napoli et al., 2008) and elongation (Ceman et al., 2003). Indeed, a majority of FMRP is associated with stalled polyribosomes (Feng et al., 1997; Stefani et al., 2004; Darnell et al., 2011) and loss of FMRP often results in increased cerebral protein synthesis rate (Osterweil et al., 2010; Qin et al., 2013; Jacquemont et al., 2018). Several high-throughput approaches have indicated that FMRP associates with thousands of mRNA targets (approximately 4–8% of all brain mRNA) with wide-ranging effects on neuronal function (Brown et al., 2001; Darnell et al., 2011; Ascano et al., 2012). Targets include a large fraction of the synaptic proteome in both pre- and post-synaptic compartments, ion channels important for regulation of cellular excitability, as well as transcription factors and chromatin-modifying proteins that can broadly affect the genetic and proteomic content of cells. FMRP can also influence cell excitability through direct protein–protein interactions with voltage- and ligand-gated ion channels (Deng and Klyachko, 2021).

Because of its ubiquitous expression and ability to regulate a large portion of the neuronal proteome, it is perhaps not surprising that loss of FMRP has far-reaching consequences on neuronal function. However, accumulating evidence suggests that neuronal hyperexcitability and network hyperactivity are important points of convergence for FXS pathophysiology (Contractor et al., 2015). In many instances, neuronal hyperexcitability is likely the direct result of loss of FMRP and its canonical role in regulating mRNA translation or ion channel function. However, a number of studies have also indicated that hyperexcitability in FXS can occur as a result of aberrant activity-dependent and/or homeostatic plasticity mechanisms, especially in early post-natal weeks when the neuronal circuits undergo immense changes owing to sensory experiences. In yet other cases, synaptic and cellular alterations that appear to promote hyperexcitability in FXS models may actually be compensatory changes that act to stabilize network activity. Loss of FMRP function is therefore likely to have multiple and sometimes even contradictory effects on circuit function, and interpreting these circuit level complexities requires an understanding of both the pleiotropic effects of *FMR1* mutation as well as the adaptive and maladaptive homeostatic responses to these primary changes. This balancing act is not unique to FXS either, as altered network and cellular homeostasis are thought to contribute to the pathogenesis of genetically-diverse forms of ASD (Bourgeron, 2015; Nelson and Valakh, 2015) as well as other neurodevelopmental and neurocognitive disorders (Frere and Slutsky, 2018; Kavalali and Monteggia, 2020). Thus, the goal of this review is to use FXS as a model for understanding the dynamic and varied processes that contribute to emergent circuit dysfunction in neuropsychiatric disorders. Below we will examine the evidence for altered excitability and plasticity in FXS models, primarily focusing on the *Fmr1* KO mouse. We will pay particular attention to the complex interplay between excitability and plasticity phenotypes, and discuss how these synaptic and circuit deficits contribute to disease-relevant behavioral phenotypes like epilepsy and sensory hypersensitivity.

### Hyperexcitable Neurons and Networks in Fragile X Syndrome

**Figure 1:**
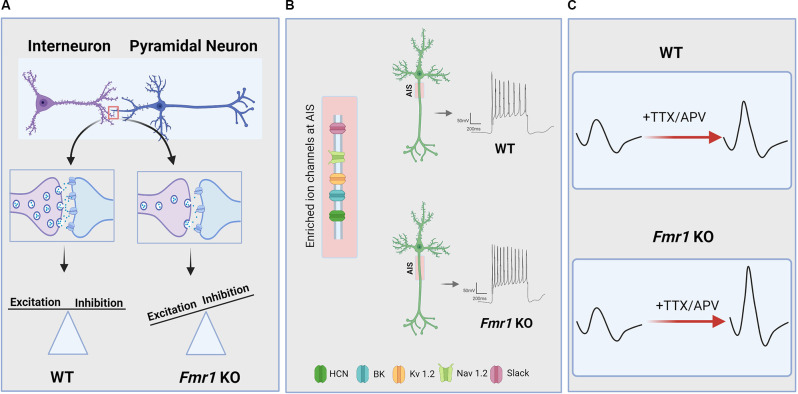
Neuronal and circuit hyperexcitability in Fragile X Syndrome (FXS). Hyperexcitability owing to loss of function of the *FMR1* gene and its protein product FMRP manifests across levels of the CNS *via* a variety of mechanisms. **(A)** Several lines of evidence indicate that disrupted excitatory/inhibitory synaptic balance due to altered activity-dependent refinement of synaptic connectivity and impaired synaptic transmission and plasticity contribute to circuit hyperexcitability in FXS. In particular, there is evidence for a reduction in inhibitory synaptic transmission in several brain regions of *FMR1* KO animals which, in addition to excessive excitatory connectivity in some cases, can result in increased E/I ratio and circuit hyperexcitability. **(B)** FMRP deficiency is also associated with dysregulated ion channel function and expression, resulting in changes to intrinsic excitability, action potential (AP) slope and duration, and increased the axon initial segment (AIS) length in some brain areas. AIS is enriched in many of the ion channels that are directly or indirectly regulated by FMRP. **(C)** Hyperexcitability in FXS can also arise from impaired homeostatic plasticity, which is an essential mechanism for maintaining basal network activity and preventing circuit hypo- or hyperexcitability. For instance, *FMR1* KO neurons exhibit dysregulated homeostatic changes to intrinsic excitability in response to activity blockade, resulting in increased AP slope and cellular hyperexcitability compared to WT neurons.

Many FXS phenotypes can be understood through the lens of neuronal hyperexcitability, with the prevalence of sensory hypersensitivity, hyperactive/aggressive behavior, epileptic seizures, and abnormal EEGs in FXS individuals and *FMR1* KO animal models confirming neuronal network hyperexcitability as a characteristic defect owing to FMRP deficiency (Musumeci et al., 2000; Berry-Kravis, 2002; Lozano et al., 2014). While hyperexcitability is observed across many cortical and subcortical brain regions, the exact mechanisms generating this phenotype appear to vary by brain region and this may have important implications for the treatment of the disorder. A wide range of studies have pointed out that the loss of FMRP disrupts innumerable signaling pathways essential for the maintenance of normal synaptic function and neuronal network stability (Bhakar et al., 2012). Hyperexcitability in FXS can be explained as a function of a number of changes, including: (1) abnormal activity-dependent refinement of synaptic connectivity leading to elevated numbers of excitatory synapses in certain neuronal populations; (2) impaired inhibitory neuron function and/or synaptic properties leading to an altered balance between excitatory and inhibitory strength (E/I imbalance); and (3) disruption in ion channel function or expression, leading to increased intrinsic excitability and altered dendritic integration. Indeed, there is evidence for changes in all of these processes in the *FMR1* deficient brain (Figure 1) and that they interact with one another in a complex fashion.

### Altered Excitatory Synaptic Function and Plasticity in FXS

One of the earliest synaptic phenotypes identified in FXS was the presence of abnormal dendritic spines, where the majority of excitatory synapses are formed in the brain. Golgi stain studies have found an overabundance of immature spines in both *FMR1* KO mice (Comery et al., 1997; Galvez and Greenough, 2005; Mckinney et al., 2005) and FXS human tissue (Hinton et al., 1991; Wisniewski et al., 1991; Irwin et al., 2001). Subsequent live-imaging experiments using two-photon microscopy have found spine density and/or shape phenotypes to be more variably expressed in FXS models, being sensitive to brain region, developmental age, and genetic background (Nimchinsky et al., 2001; Meredith et al., 2007; Cruz-Martín et al., 2010; Harlow et al., 2010; Pan et al., 2010; He and Portera-Cailliau, 2013). However, these live-imaging studies also highlighted the fact that, regardless of overall number or shape differences, dendritic spines in *FMR1* KO animals exhibit atypical dynamics and were much less sensitive to changes in activity levels or sensory experience (Wisniewski et al., 1991; Antar et al., 2006; Goel et al., 2006; Pan et al., 2010). Thus, loss of FMRP leads to impaired activity-dependent changes to spine structure and number, resulting in abnormal synaptic maturation, stabilization, and/or elimination (Comery et al., 1997; Cruz-Martín et al., 2010; Pfeiffer et al., 2010).

Consistent with anatomical studies of spine dynamics, electrophysiological experiments have found differences in excitatory synaptic function in *FMR1* KO models, once again with an emphasis on disrupted activity-dependent modifications (Sidorov et al., 2013). Early studies found no alteration to basal synaptic transmission or long-term potentiation (LTP) in the hippocampus of *FMR1* KO mice (Godfraind et al., 1996; Paradee et al., 1999), although subsequent studies have found subtle LTP deficits in the hippocampus (Lauterborn et al., 2007; Hu et al., 2008) and other brain regions (Meredith et al., 2007; Koga et al., 2015). The most prominent synaptic plasticity phenotype observed in *FMR1* KO models is excessive group 1 metabotropic glutamate receptor (mGluR1/5)-dependent long-term depression (LTD) at excitatory synapses (Huber et al., 2002; Koekkoek et al., 2005; Hou et al., 2006; Till et al., 2015). Expression of mGluR-LTD in the mature brain is mediated *via* post-synaptic internalization of AMPA receptors (Snyder et al., 2001; Gladding et al., 2009), which is stabilized by rapid *de novo* synthesis of proteins from pre-existing, dendritically-localized mRNA (Huber et al., 2000). Interestingly, FMRP itself is one of the proteins synthesized by mGluR 1/5 activation (Weiler et al., 1997; Antar et al., 2004; Hou et al., 2006). These findings, coupled with FMRP’s role in repressing activity-dependent protein synthesis, have led to the idea that FMRP acts as a negative feedback regulator to limit mGluR-mediated protein synthesis (Bear et al., 2004). Consistent with this notion, basal protein synthesis rates are elevated in the hippocampus of *FMR1* KO mice and mGluR5-mediated increases in protein synthesis are occluded in slices from *FMR1* animals (Todd et al., 2003; Osterweil et al., 2010). Similarly, mGluR-LTD is not only exaggerated in *FMR1* KO animals but it no longer requires new protein translation (Hou et al., 2006; Nosyreva and Huber, 2006). Importantly, post-natal re-expression of FMRP in *FMR1* KO slices can restore normal levels of mGluR-LTD (Zeier et al., 2009). It is unclear how exaggerated mGluR-LTD contributes to neuronal hyperexcitability in FXS, as enhanced synaptic depression at excitatory synapses would likely act to decrease excitatory drive onto neurons. However, it may be more informative to view mGluR-LTD as a sensitive functional read-out of mGluR-stimulated protein synthesis in dendrites, which has a number of consequences in addition to LTD that could directly contribute to neuronal hyperexcitability, such as facilitating the persistence of LTP (LTP priming; Raymond et al., 2000) and inducing prolonged epileptiform discharges (Bianchi et al., 2009). Indeed, mGluR-mediated priming of LTP (Auerbach and Bear, 2010) and mGluR-induced epileptiform activity (Chuang et al., 2005; Zhao et al., 2011) are enhanced and/or uncoupled from activity-dependent protein synthesis in the hippocampus of *FMR1* KO mice, both of which would act to increase circuit excitability.

#### Disrupted Critical Period Plasticity and Synaptic Refinement

Activity-dependent synaptic modification is a crucial step in normal development (Faust et al., 2021). As FMRP is highly expressed during early life critical periods (Till, 2010; Bonaccorso et al., 2015; Gholizadeh et al., 2015), loss of FMRP may lead to altered excitatory synaptic development, which in turn could contribute to hyperexcitability phenotypes in FX. There is indeed evidence for deficient or disrupted critical period plasticity in *FMR1* KO mice. Whole cell recordings from layer 4 stellate cells in barrel cortex slices from juvenile *FMR1* KO mice have found increased persistence of silent synapses, those containing NMDAR but not AMPAR currents, at later developmental time points compared to wild-type (WT) animals, which corresponded with a shift in the temporal window for LTP induction at these synapses (Harlow et al., 2010). Intracortical connections in the barrel cortex of *FMR1* KO mice were also shown to exhibit abnormal development in a temporally restricted manner (Bureau et al., 2008). In the auditory system, passive exposure to tones during the auditory critical period results in shifts to the tonotopic map of sound frequency representation in the auditory cortex (Zhang L. I. et al., 2001). This critical period auditory plasticity was absent in *FMR1* KO mice (Kim et al., 2013), potential due to impaired stabilization of LTP at auditory thalamocortical synapses at this development time-point (Yang et al., 2014). It is also important to note that FMRP’s role in circuit development is not restricted to the cortex, as post-synaptic reduction of FMRP in chick auditory brainstem *via* *in utero* electroporation leads to a delay in dendrite branch retraction and the prevention of presynaptic endbulb development (Wang et al., 2018). These studies indicate that FMRP is important for defining the critical window for neuronal circuit refinement during development.

How might dysregulated critical period plasticity result in hyperexcitable circuits? A central mechanism for developmental refinement of neural circuits is synaptic pruning, i.e., the activity-dependent elimination of synapses (Sakai, 2020; Faust et al., 2021). Several studies have indicated that synapse elimination is disrupted in *FMR1* KO animals. In drosophila, loss of FMRP has been shown to alter dendritic complexity and synapse growth at glutamatergic neuromuscular junctions (Zhang Y. Q. et al., 2001) and in the central nervous system (Pan et al., 2004; Kennedy et al., 2020). Dual patch experiments have found evidence for overconnectivity of excitatory neurons in acute slices from the somatosensory cortex of *FMR1* KO mice (Patel et al., 2014). Interestingly, this hyperconnectivity phenotype was not due to increased development of synaptic connections in *FMR1* KO mice but rather to a failure in activity-dependent synaptic elimination between 3 and 5 weeks postnatal. Similar synaptic pruning deficits have been observed in hippocampal slice cultures, where it was shown that synapse elimination *via* the activity-dependent transcription factor MEF2 is absent in slices from *FMR1* KO mice (Pfeiffer et al., 2010). Importantly, acute post-synaptic re-expression of FMRP was able to restore MEF2-dependent synapse elimination in KO slices, suggesting FMRP regulates excitatory synapse elimination in a cell-autonomous manner. FMRP was subsequently shown to regulate MEF2-dependent synapse elimination *via* PP2A-mediated dephosphorylation of the ubiquitin E3 ligase murine double minute-2 (Mdm2), which promotes the degradation of the synaptic scaffolding protein PSD-95 (Tsai et al., 2017). Most recently, post-synaptic loss of FMRP in the somatosensory cortex has been shown to result in impaired activity-dependent development of callosal inputs, resulting in increased local intracortical connectivity but impaired long-range cortical-cortical connections (Zhang et al., 2021).

FMRP is also expressed in pre-synaptic terminals (Christie et al., 2009), and pre-synaptic loss of FMRP may regulate excitatory post-synaptic development as well (Antar et al., 2006). Indeed, studies using mosaic deletion of *Fmr1* in hippocampal slice culture found that pre-synaptic loss of FMRP was sufficient to increase synaptic connectivity while postsynaptic deletion did not alter connection probability (Hanson and Madison, 2007). While the mechanisms governing abnormal pre-synaptic development with *FMR1* deletion remain to be fully elucidated, there is intriguing evidence that FMRP can regulate pre-synaptic transmitter release via direct modulation of ion channel function independent of its role in translation regulation (see Section “Ion Channel Dysregulation and Altered Intrinsic Excitability in FXS”; Ferron et al., 2014; Myrick et al., 2015). Whether pre- or post-synaptic in nature, deficient synapse elimination has the potential to lead to hyperexcitability in mature circuits. For instance, *in vivo* recordings from the lateral superior olive (LSO), an auditory brainstem area important for sound localization, found evidence for increased sound-evoked activity and hyperexcitability at the population level in *FMR1* KO mice (Garcia-Pino et al., 2017). Parallel whole cell slice recordings found no difference in the properties of individual excitatory or inhibitory synapses in this region, but rather that hyperexcitability was the result of an increased number of excitatory connections converging onto individual LSO neurons. Ultrastructure analysis in the somatosensory cortex shows that loss of FMRP results in a three-fold increase in multiply-innervated spines, leading to increased single-spine excitation that promotes circuit hyperexcitability (Booker et al., 2019). Thus, hyperexcitable circuits in FXS could be due in part to failures of synaptic pruning during development as a consequence of dysregulated experience-dependent plasticity.

### Altered Inhibitory Neuron Function in FXS

Efficient information processing in neural circuits requires a tightly regulated balance between excitatory and inhibitory activity (E/I balance; Haider et al., 2006; Shew et al., 2011; Yizhar et al., 2011). As discussed above, loss of FMRP alters the development and function of excitatory synapses in a number of ways that could affect neuronal excitability. FMRP is also broadly expressed in GABAergic neurons (Feng et al., 1997; Olmos-Serrano et al., 2010) and many lines of evidence point to altered inhibitory neuronal function in FXS as well. *FMR1* KO mice have reduced levels of several GABA_A_ receptor subunits, the major fast-acting inhibitory ionotropic receptor in the brain, at both the mRNA (D’hulst et al., 2006; Gantois et al., 2006) and protein levels (El Idrissi et al., 2005; Gantois et al., 2006; Curia et al., 2009). Pre-synaptically, expression of the rate-limiting GABA synthesizing enzyme glutamic acid decarboxylase (GAD) has been shown to be reduced in *FMR1* KO mice (Olmos-Serrano et al., 2010), although other studies have found increased GAD65/67 expression in some brain regions (El Idrissi et al., 2005). Down-regulation of GABA_A_ receptors and GAD have also been observed in the drosophila fly model of FXS (Gatto et al., 2014). Anatomical defects in GABAergic and/or glycinergic neurons have been observed in the cortex (Selby et al., 2007) and brainstem (Mccullagh et al., 2017) of *FMR1* KO mice. *In vivo* imaging studies have found impaired sensory-evoked activity in inhibitory neuron populations in the cortex of *FMR1* KO mice as well (Goel et al., 2018). Human PET imaging studies have found evidence for diminished GABA_A_ receptor binding in the brains of FXS individuals (D’hulst et al., 2015). Electroencephalography (EEG; Ethridge et al., 2017; Wang et al., 2017) and transcranial magnetic stimulation studies (TMS; Morin-Parent et al., 2019) have found indirect evidence for reduced inhibition in humans with FXS in the form of altered neuronal oscillations and reduced short-interval suppression of TMS-evoked potentials, which both depend on local intracortical inhibition (Kujirai et al., 1993; Chen et al., 2008; Cardin et al., 2009; Sohal et al., 2009). Thus, there is general agreement that *FMR1* mutation results in a broad dampening of GABAergic inhibition in the brain which could lead to hyperexcitable networks (Figure 1A). However, it is also clear that the concept of a single E/I balance is overly simplistic, as there are different sources of inhibition within a single microcircuit that target distinct cellular compartments and affect different aspects of neuronal function (O’donnell et al., 2017). It is also likely that disruptions to excitatory synaptic function in FXS can evoke changes to inhibitory transmission and *vice versa*. Thus, it is important to understand the precise manner in which inhibitory synaptic and circuit function are altered in FXS in order to fully understand the consequences of these changes on network excitability and information processing.

#### Deficient GABAergic Transmission in FMR1 KO Models

Electrophysiological studies have found evidence for reduced GABAergic inhibition onto excitatory principal cells in *FMR1* KO animals in a variety of brain areas, albeit with region-specific differences. Consistent with evidence for changes to the pre- and post-synaptic machinery for GABAergic signaling in FXS, both the frequency and amplitude of spontaneous and miniature inhibitory post-synaptic potentials (sIPSCs, mIPSCs) are reduced in the amygdala of adult (Olmos-Serrano et al., 2010) and juvenile (Vislay et al., 2013) *FMR1* KO mice. Conversely, GABAergic inhibition was found to be enhanced in the striatum of adult *FMR1* mice *via* increased pre-synaptic transmitter release (Centonze et al., 2008). Basal GABAergic transmission was not altered in layer 2/3 pyramidal neurons in the somatosensory cortex of *FMR1* KO mice, but mGluR-mediated activation of low-threshold spiking (LTS) interneurons was deficient, resulting in reduced activity-dependent inhibition (Paluszkiewicz et al., 2011). mGluR-dependent decreases in inhibitory function *via* retrograde endocannabinoid signaling have also been observed in the hippocampus (Zhang and Alger, 2010), striatum (Maccarrone et al., 2010), and cortex (Rio et al., 2018) of *FMR1* KO mice, once again highlighting the role of FMRP in mGluR-dependent plasticity. Action potential evoked feed-forward inhibitory input to the CA1 region of the hippocampus is reduced in *FMR1* KO mice in an input-specific manner (Wahlstrom-Helgren and Klyachko, 2015, 2016). Decreased feed-forward inhibition onto excitatory neurons has also been observed in the lateral amygdala (Svalina et al., 2021) and the somatosensory cortex (Antoine et al., 2019; Domanski et al., 2019) of *FMR1* KO mice. Loss of feedforward inhibition is associated with marked changes in E/I balance, increased spike probability, and reduced spike precision, all of which are likely to contribute to circuit hyperexcitability and impaired information processing in these areas. However, it should also be noted that, in some cases, decreased inhibitory synaptic transmission and enhanced E/I ratio in *FMR1* KO mice may actually act to stabilize circuit excitability. For instance, Antoine and colleagues found that *FMR1* KO mice exhibited reduced feedforward inhibition onto layer 2/3 pyramidal neurons in the somatosensory cortex but that this reduction in inhibitory conductance was not associated with increased whisker-evoked spiking activity in these neurons (Antoine et al., 2019). Instead, modeling experiments suggested that rather than promoting network hyperexcitability, altered E/I balance in layer 2/3 neurons may actually reflect a homeostatic process to maintain stable synaptic drive.

GABA_A_ receptors not only mediate fast-acting, synapse-specific phasic inhibition but in some brain areas can also mediate slower, sustained tonic inhibition involving extrasynaptic GABA_A_ receptors (Farrant and Nusser, 2005). Both phasic and tonic inhibition were shown to be deficient in the amygdala of *FMR1* KO animals (Olmos-Serrano et al., 2010; Martin et al., 2014), while tonic but not phasic inhibition was disrupted in the subiculum (Curia et al., 2009). Increased tonic inhibition did not alter overall synaptic conductance or E/I balance in *FMR1* KO animals, but it impaired the timing between feedforward excitation and inhibition, and this disruption in the temporal precision of stimulus-evoked E/I balance may contribute to hyperexcitability (Martin et al., 2014). Acute treatment of *FMR1* KO mice with gaboxadol, a GABA_A_ receptor agonist selective for extrasynaptic receptors mediating tonic currents, rescues hyperexcitability of amygdala principal neurons and rescued certain behavioral phenotypes in *FMR1* KO mice, suggesting reduced tonic GABAergic inhibition in the amygdala contributes to hyperexcitability phenotypes in FXS (Olmos-Serrano et al., 2010, 2011). In fact, recently completed phase 2 clinical trials investigating the use of gaboxadol to treat FXS have shown promising results (Budimirovic et al., 2021). Thus, circuit hyperexcitability in many brain regions of *FMR1* KO animals is likely due in part to decreased basal GABAergic transmission and/or altered activity-dependent changes to inhibitory drive onto excitatory neurons.

GABA_B_ receptors are metabotropic receptors that can regulate cellular excitability both pre- and post-synaptically by hyperpolarizing neurons and limiting neurotransmitter release *via* activation of inwardly-rectifying K^+^ channels and inhibition of voltage-gated Ca^2+^ channels (Pinard et al., 2010). Due to their broad regulation of pre- and post-synaptic excitability, and specifically, their potential to reduce glutamate release and subsequent downstream activation of mGluR5, GABA_B_ agonists like arbaclofen have been explored as a potential FXS therapy (Berry-Kravis et al., 2012, 2017). Arbaclofen has indeed been shown to normalize protein synthesis rates as well as a variety of physiological and behavioral phenotypes in *FMR1* KO mice (Henderson et al., 2012; Silverman et al., 2015; Sinclair et al., 2017a). However, clinical trials with arbaclofen have proved unsuccessful (Berry-Kravis et al., 2017) and recent animal studies found that chronic baclofen treatment can actually result in exacerbation of FXS phenotypes, potentially due to drug tolerance development (Zeidler et al., 2018). Drug tolerance development may also limit the effectiveness of other potential FXS therapies, like mGluR5 inhibitors (Stoppel et al., 2021). It is also important to note that GABA_B_ receptors are also expressed at pre-synaptic inhibitory terminals. Indeed, decreased feedforward inhibition in the hippocampus on *FMR1* KO mice was shown to be driven by increased pre-synaptic GABA_B_ receptor signaling, leading to reduced GABA release (Wahlstrom-Helgren and Klyachko, 2015). Thus, treatments that enhance GABA_B_ signaling may act to promote FXS hyperexcitability phenotypes in some cases as well.

Finally, in addition to changes in GABAergic synaptic transmission, altered excitatory drive onto inhibitory neurons has been observed in *FMR1* KO animals. Dual patch clamp recordings from directly coupled excitatory and inhibitory neurons in the somatosensory cortex of juvenile *FMR1* KO mice have shown that there is reduced feedforward excitatory input onto layer 4 fast-spiking (FS) inhibitory neurons (Gibson et al., 2008). This decrease in feedforward excitation was also associated with an increase in persistent UP states in both in slice (Gibson et al., 2008) and *in vivo* (Hays et al., 2011), which are brief periods of persistent depolarized firing states in neurons that are indicative of increased network excitability. Transient increases in UP states were also observed in layer 2/3 somatosensory cortical neurons of *FMR1* KO mice during the critical period (Goncalves et al., 2013). Prolonged UP states in *FMR1* KO mice were rescued by genetic reduction or pharmacological inhibition of mGluR5, suggesting this hyperexcitability phenotype may be related to altered glutamatergic signaling (Hays et al., 2011). Interestingly, reduced excitatory input onto FS inhibitory neurons appears to be due to pre-synaptic loss of FMRP, as selective deletion of *Fmr1* in excitatory neurons resulted in prolonged UP states while selective deletion in inhibitory neurons had no effect (Hays et al., 2011). Indeed, mosaic deletion of *Fmr1* demonstrated that pre-synaptic loss of FMRP in the somatosensory cortex resulted in a specific reduction in presynaptic glutamate release onto post-synaptic inhibitory neurons without affecting excitatory-excitatory connections, indicative of target-specific function for presynaptic FMRP (Patel et al., 2013). Coupled with the evidence for deficits in pruning at excitatory-excitatory connections discussed above (Hanson and Madison, 2007; Pfeiffer et al., 2010; Patel et al., 2014), these studies indicate that pre- vs. post-synaptic loss of FMRP may differentially regulate excitatory and inhibitory synaptic connectivity, resulting in an imbalance to E/I connectivity and network hyperexcitability.

#### Cell-Type-Specific Changes in Inhibitory Neuron Function

Inhibitory interneurons consist of genetically and anatomically diverse cell populations that subserve distinct roles in circuit function. Thus, understanding the consequences of altered inhibitory function in FXS requires understanding the cell-type specific effects of inhibitory neuron sub-populations. The three most common genetically-defined interneuron classes in the cortex are parvalbumin positive (PV), somatostatin positive (SST), and vasoactive intestinal peptide positive (VIP) interneurons (Defelipe et al., 2013). PV neurons largely overlap with FS basket cells that provide strong perisomatic inhibition to regulate excitatory neuron output. Anatomical studies have found a pronounced decrease in PV neuron density in the cortex of *FMR1* KO animals (Selby et al., 2007), and *in vivo* Ca^2+^ imaging from genetically-identified PV neurons found reduced sensory-evoked activity in PV neuron populations in the visual cortex of *FMR1* KO mice, which corresponded with impaired perceptual learning (Goel et al., 2018).

Fast-spiking PV neurons play an integral role in regulating the synchronization of cortical circuits, particularly in the high frequency gamma range (Cardin et al., 2009; Sohal et al., 2009). Interestingly, EEG studies have observed increased cortical gamma oscillation in FXS individuals (Ethridge et al., 2017; Wang et al., 2017) as well as *FMR1* KO mice (Lovelace et al., 2018) and rats (Kozono et al., 2020). Similar changes in gamma power are observed in acute cortical slices from *FMR1* KO animals as well, suggesting observed EEG abnormalities are driven in part by local alterations in neocortical circuits (Goswami et al., 2019). Interestingly, EEG alterations in *FMR1* KO mice can be rescued by genetic reduction of matrix metallopeptidase 9 (MMP9), an enzyme involved in the degradation of perineuronal nets (PPNs) which preferentially stabilize synaptic connections with PV neurons (Lovelace et al., 2016; Wen et al., 2018) and whose mRNA has been shown to be a target of FMRP (Janusz et al., 2013). As PV neurons strongly overlap with electrophysiologically characterized FS interneurons, reduction in sensory-evoked PV activity and altered EEG oscillations may be due to deficient intracortical excitatory input onto FS interneurons described above (Gibson et al., 2008), potentially as a consequence of altered MMP9 activity (Wen et al., 2018). Consistent with this notion, forebrain deletion of *FMR1* specifically in excitatory neurons recapitulates increased MMP9 activity and a majority of EEG deficits seen in global *FMR1* KO mice (Lovelace et al., 2020). Recent studies have demonstrated that minocycline treatment, an FDA-approved antibiotic that can inhibit MMP9 activity, reverses electrophysiological and/or behavioral disturbances in *FMR1* KO mice (Bilousova et al., 2009; Lovelace et al., 2020), drosophila FXS models (Siller and Broadie, 2011), and FXS individuals (Leigh et al., 2013).

Beyond PV neurons, the function of other inhibitory interneuron subtypes in FXS has been less well-characterized. Slice recordings from the somatosensory cortex found impaired mGluR-dependent activation of SST-expressing LTS neurons that target distal dendrites to regulate the integration of synaptic input. This reduced activity-dependent inhibition onto excitatory neurons resulted in altered cortical synchronization in the form of elevated low-frequency theta oscillations (Paluszkiewicz et al., 2011). Thus, loss of FMRP can have distinct effects on network function *via* differential regulation of distinct inhibitory interneurons subtypes. VIP interneurons are less numerous than PV and SST neurons but can have a broad impact on cortical circuit function *via* targeting of other interneuron subtypes, forming a disinhibitory circuit (Pfeffer et al., 2013). To our knowledge, no studies have directly assessed VIP interneuron function in FXS models to date.

#### Altered GABAergic System Development in FXS

The above studies suggest that abnormal inhibitory neuron function in *FMR1* KO animals results from a combination of decreased inhibitory drive onto excitatory neurons and decreased excitatory drive onto inhibitory neurons. These changes are associated with marked changes in E/I balance and neuronal processing in diverse brain regions. As discussed in section “Altered Excitatory Synaptic Function and Plasticity”, FMRP is an important regulator of activity-dependent refinement of excitatory synaptic function. GABAergic transmission also plays a critical role during early brain development, where it acts *via* paracrine, non-synaptic signaling to depolarize neurons due to high intracellular Cl^−^ concentration at this developmental time point (Represa and Ben-Ari, 2005). Recent studies have demonstrated that *Fmr1* deletion delays the developmental switch in GABA polarity from depolarizing to hyperpolarizing in the cortex (He et al., 2014) and hippocampus (Tyzio et al., 2014) due to the developmentally elongated expression of the juvenile Cl^−^ transporter NKCC1. No differences in the expression level of the adult Cl^−^ transported KCC2 were observed at any post-natal timepoint in *FMR1* KO mice (He et al., 2014). This delayed maturation of GABAergic signaling is likely to have a profound impact on synaptic and circuit development, similar to altered critical period plasticity of excitatory synaptic function observed in *FMR1* KO animals. Indeed, it was recently shown that inhibiting NKCC1 with the FDA-approved drug bumetanide during the somatosensory critical period corrects the development of thalamocortical excitatory synapses and altered whisker-evoked receptive fields in adult *FMR1* KO mice (He et al., 2018).

Inhibitory synapse formation is also developmentally regulated, characterized by a rapid increase in synapse number and maturation around the end of the 4th postnatal week (Micheva and Beaulieu, 1996; Oh and Smith, 2019). This maturation of cortical GABAergic neurons, particularly PV interneurons, is thought to contribute to the closure of developmental critical periods (Pizzorusso et al., 2002; Balmer et al., 2009). Intriguingly, this inhibitory maturation and critical period closure also coincide with the formation of PNNs. Indeed, there is evidence that PNN-dependent stabilization of PV neuron function directly contributes to the closure of critical period plasticity windows (Lee et al., 2017; Lensjo et al., 2017; Murase et al., 2017). Thus, it is possible that the abnormal development of PNNs and PV cells observed in the cortex of *FMR1* KO animals (Selby et al., 2007; Wen et al., 2018) may underly delayed or impaired critical period plasticity seen at excitatory synapses in these animals (Harlow et al., 2010; Kim et al., 2013), although this hypothesis remains to be explicitly tested. Taken together, these studies indicate that altered excitatory circuit development in FXS may be due in part to GABAergic defects.

### Ion Channel Dysregulation and Altered Intrinsic Excitability in FXS

FMRP acts through a variety of direct and indirect mechanisms to regulate the expression and function of multiple ion channels in the brain, including: voltage-gated Na^+^, K^+^, and Ca^2+^ channels; hyperpolarization-activated cyclic nucleotide-gated (HCN) channels; and small- and big- conductance Ca^2+^- activated (SK, BK) K^+^ channels (Deng and Klyachko, 2021; Figure 1B). Several ion channels have been identified as FMRP targets (Darnell et al., 2011), suggesting that *FMR1* deletion can influence cellular excitability through its canonical role as a translation regulator. These include Kv3.1 (Strumbos et al., 2010), Kv4.2 (Lee et al., 2011), and HCN1 channels (Brager et al., 2012). Interestingly, FMRP can also modulate the function of several ion channels *via* direct protein-protein interactions, including the Na^+^-activated K^+^ channel Slack (Brown et al., 2010), BK (Deng et al., 2013; Myrick et al., 2015), and SK (Deng et al., 2019) channels. Finally, loss of FMRP can influence cellular excitability indirectly through dysregulation of cell signaling pathways (Chuang et al., 2005; Zhao et al., 2011; Deng and Klyachko, 2016a). Thus, ion channel function is altered through a variety of mechanisms in FXS and this is likely to influence a wide-range of neuronal processes, including intrinsic excitability, neurotransmitter release, and dendritic integration.

### Increased Intrinsic Excitability in *Fmr1* KO Models

Several studies have demonstrated increased intrinsic excitability across brain regions in *FMR1* KO animals, although as in the case of synaptic disturbances, the effects vary across brain regions. In addition to reduced feedforward excitation onto FS interneurons, a modest increase in the excitability of layer 4 principal neurons in the somatosensory cortex is observed in *FMR1* KO mice as a result of increased membrane capacitance and input resistance (Gibson et al., 2008; Domanski et al., 2019). Several studies have observed increased stimulus-evoked action potential (AP) generation in layer 5 cortical pyramidal neurons, which appears to depend on altered mGluR activity and downstream signaling components (Hays et al., 2011; Osterweil et al., 2013; McCamphill et al., 2020). However, whole-cell recordings from layer 5 pyramidal cells in entorhinal (Deng et al., 2013) and somatosensory (Zhang et al., 2014) cortex found no difference in intrinsic parameters in these neurons, suggesting this stimulus-evoked hyperexcitability may be synaptically generated. Intrinsic hyperexcitability in the entorhinal (Deng and Klyachko, 2016a) and prefrontal (Routh et al., 2017) cortex of *FMR1* KO mice was shown to depend on increased non-inactivating persistent Na^+^ current (I_NaP_). Interestingly, increased I_NaP_ current in the entorhinal cortex was not due to direct modulation of ion channel expression or function by FMRP, but rather through exaggerated mGluR5 signaling (Deng and Klyachko, 2016a). Few studies have directly assessed the intrinsic properties of inhibitory neurons in *FMR1* KO models, but those that have found no differences (Gibson et al., 2008).

FMRP has been shown to directly regulate the expression of the voltage-gated K^+^ channel Kv3.1 (Darnell et al., 2011), whose experience-dependent expression gradients are altered in the medial nucleus of the trapezoid body (MNTB) of *FMR1* KO mice (Strumbos et al., 2010). Abnormal expression of Kv3.1 in the MNTB leads to faster repolarization and higher firing rates, indicative of hyperexcitability (El-Hassar et al., 2019). The MNTB is an essential component of the sound localization circuitry of the auditory brainstem, which requires rapid temporal processing of incoming sound information to compute interaural cue differences (Grothe et al., 2010). Thus, tight regulation of neuronal excitability is essential for allowing MNTB principal cells to fire at high rates with high temporal fidelity. Slack channels account for a major component of the total K^+^ current in principal neurons of MNTB and some of the first evidence for direct FMRP-ion channel interactions was observed for Slack channels in the MNTB (Brown et al., 2010). Loss of FMRP reduces Slack currents, thereby increasing neuronal excitability and reducing temporal precision of spiking. MNTB principal neurons send glycinergic projections to the LSO, which uses a precise comparison of inhibitory inputs from the MNTB and excitatory inputs from the cochlear nucleus to compute interaural level differences (Park et al., 1996). Interestingly, hyperexcitability is also observed in principal cells of LSO in *FMR1* KO mice, but in the absence of intrinsic property differences (Garcia-Pino et al., 2017). Rather, LSO hyperexcitability was found to be caused by increased excitatory synaptic connectivity from cochlear nucleus afferents, while inhibitory inputs from the MNTB were unchanged. As tightly regulated E/I balance is essential in this sound localization circuit, it is tempting to speculate that altered excitatory connectivity in the LSO may arise to compensate for hyperexcitable inhibitory inputs from the MTNB or* vice versa*.

#### Effect of Ion Channel Dysregulation on Synaptic Function in FXS

Ion channels are not only involved in setting AP threshold and firing rate but can affect a variety of synaptic processes as well. For instance, BK channels play a critical role not only in regulating neuronal excitability but also in modulating AP duration and neurotransmitter release (Salkoff et al., 2006). FMRP has been shown to regulate BK channel conductance and expression and loss of this regulation in *FMR1* KO mice leads to decreased BK activity, resulting in AP broadening, which in turn leads to elevated presynaptic Ca^2+^ influx, increased glutamate release, and alterations to short-term pre-synaptic plasticity (Deng et al., 2013; Zhang et al., 2014; Myrick et al., 2015). Genetic upregulation of the BK β4 subunit rescues the observed excitability and synaptic defects (Deng and Klyachko, 2016b). Moreover, treatment of *FMR1* KO mice with the BK channel open BMS-204351 corrected a variety of hyperexcitability and behavioral phenotypes, suggesting BK channels may be a valuable therapeutic target to treat FXS (Zhang et al., 2014; Carreno-Munoz et al., 2018). FMRP-dependent AP broadening is observed in both hippocampal and cortical pyramidal neurons and has a cell-autonomous pre-synaptic origin (Deng et al., 2013). Future work must determine how FMRP-BK channel interaction may contribute to alterations in excitatory-excitatory and/or excitatory-inhibitory connectivity observed in the hippocampus and cortex of *FMR1* KO mice that has been shown to depend on pre-synaptic loss of FMRP as well (Hanson and Madison, 2007; Patel et al., 2013). FMRP has also been shown to regulate pre-synaptic GABA release in cerebellar basket cells *via* modulation of the expression and activity of Kv1.2 (Yang et al., 2020). Loss of FMRP-mediated regulation of Kv1.2 leads to enhanced pre-synaptic Ca^2+^ influx and excessive GABA release onto Purkinje neurons. While these changes would appear to counteract hyperexcitability, Purkinje cells themselves are inhibitory, so the net effect of these changes would be disinhibition of Purkinje targets and thus still promote circuit hyperexcitability.

Ion channel regulation is not only important for pre-synaptic transmitter release, but also for the dendritic integration of post-synaptic signals (Stuart and Spruston, 2015). One of the first channels identified as a target of FMRP was the voltage-gated K^+^ channel Kv4.2, whose expression was shown to be elevated in the hippocampus *FMR1* KO mice (Lee et al., 2011). Kv4.2 is a dendritic localized channel that mediates A-type currents that act to suppress AP-backpropagation into dendrites, which is important for modulating LTP induction (Chen et al., 2006). Thus, increased Kv4.2 expression may contribute to elevated thresholds for LTP induction in *FMR1* KO animals (Lauterborn et al., 2007; Meredith et al., 2007). However, other studies have found evidence for reduced Kv4.2 levels in *FMR1* KO mice (Gross et al., 2011) and dendritic recordings from hippocampal pyramidal neurons found decreased A-current in *FMR1* KO mice, which was associated with enhanced rather than impaired LTP induction (Routh et al., 2013). The discrepancies between these studies remain unclear, as similar biochemical techniques and LTP induction protocols were used. One potential explanation is a difference in the properties of more proximal synapses near the soma compared to distal dendritic synapses examined by Routh and colleagues. It will be important for future studies to examine both somatic and dendritic excitability in *FMR1* KO animals in combination with plasticity levels. A recent study has added another element to these contrary findings by demonstrating that FMRP can also directly interact with Kv4 channels to change their gating properties, resulting in reduced cellular excitability and increased LTP thresholds in cerebellar granule cells (Zhan et al., 2020). Importantly, reintroduction of an FMRP fragment that can bind Kv4 into *FMR1* KO mice restored deficits in mossy fiber LTP induction and behavioral hyperactivity assessed *via* open field test (Zhan et al., 2020).

HCN channels are cation permeable channels that underly the hyperpolarization-activated inward current (I_h_) that plays a crucial role in setting resting membrane potential and dendritic excitability (Shah, 2014). HCN1-subunit expression and dendritic I_h_ are elevated in CA1 pyramidal neurons of *FMR1* KO mice, resulting in decreased input resistance and reduced temporal summation (Brager et al., 2012). Conversely, in layer 4 stellate and layer 5 pyramidal cells, HCN1 expression and dendritic I_h_ are reduced, leading to increased dendritic gain and sensory hyperexcitability (Zhang et al., 2014). Interestingly, this cell-type-specific bidirectional regulation of HCN channels may be the result of a cell-autonomous protein-protein interaction between FMRP and HCN, providing a potential mechanism for cell-type-specific differences in *FMR1* deletion (Brandalise et al., 2020). L-type voltage-gated Ca^2+^ channels (VGCCs) are another class of ion channels important for dendritic excitability and the mRNA for several VGCCs have been shown to be targets of FMRP (Chen et al., 2003; Darnell et al., 2011). Interestingly, despite being an FMRP target, expression of Cav1.3 is downregulated in the cortex and cerebellum of *FMR1* KO mice (Chen et al., 2003), and reduced expression of L-type VGCCs is associated with impaired spike-timing-dependent-plasticity (Meredith et al., 2007). While FMRP has been shown to predominantly suppress mRNA translation, there is evidence that FMRP can promote the translation of certain mRNA transcripts (Bechara et al., 2009; Fahling et al., 2009; Gross et al., 2011). Alternatively, reduced VGCC expression in juvenile and adult *FMR1* KO animals could be a compensatory change, as it has been shown that there is increased Ca^2+^ influx through L-type VGCCs in neural progenitor cells from *FMR1* KO mice and FXS human-derived pluripotent stem (iPS) cells (Danesi et al., 2018). Together, these studies indicate the wide-ranging effects that dysregulated ion channel function can have on cellular, synaptic, and circuit properties in FXS models.

## Homeostatic Plasticity in FXS

A confluence of molecular, synaptic, and cellular perturbations contribute to the generation of circuit hyperexcitability in FXS. Some of these disruptions are likely due to abnormal embryonic and early post-natal development of brain circuits, while others appear to be due to persistent loss of FMRP function in adulthood. Because FMRP is involved in a variety of neuronal processes across developmental time-points, it is also important to consider the array of compensatory mechanisms utilized by the brain to maintain optimal activity ranges and circuit stability when attempting to elucidate the consequences of *FMR1* deletion. This is complicated by the fact that FMRP is important for many forms of activity-dependent plasticity as well, and recent evidence has highlighted the role of FMRP in regulating homeostatic plasticity both during development and in the mature brain. In this section, we will review recent findings of how FMRP contributes to homeostatic plasticity and how the loss of this regulation contributes to hyperexcitability phenotypes in FXS.

### Homeostatic Mechanisms for Maintaining Circuit Stability

Sensory acquisition in the brain begins as early as the fetal stage and occurs throughout the life of an individual (Partanen et al., 2013). Sensory experience and learning process tend to destabilize the associated neuronal circuit, which is part of a normal plasticity mechanism (Beston et al., 2010; Morgan et al., 2019). However, in order to regain circuit stability, such destabilizing forces need to be balanced by a counteracting process such as homeostatic plasticity. Information storage in neural circuits relies on Hebbian forms of synaptic plasticity, which involve activity-dependent changes in synaptic strength owing to LTP and LTD. These activity-dependent changes in synaptic strength depend on the precisely correlated firing of pre- and post-synaptic neurons. After the onset of LTP induction, the potentiated synapses enter a positive feedback loop, leading to continuous synaptic strengthening and circuit hyperexcitability (Turrigiano and Nelson, 2000; Turrigiano, 2008; Vitureira and Goda, 2013). On the other hand, induction of LTD enforces activity-dependent weakening of synapses and continuous LTD would lead to eventual silencing of synapses (Collingridge et al., 2010). Therefore, in the absence of mechanisms that can attenuate the hypo- or hyperexcitability owing to uncontrolled LTD or LTP, respectively, robust destabilizing forces in a circuit could pose devastating consequences on network activity (Abbott and Nelson, 2000). Because Hebbian plasticity requires FMRP-dependent protein synthesis (Shang et al., 2009; Sidorov et al., 2013; Guo et al., 2016), homeostatic plasticity may also require FMRP and a deficit of homeostatic plasticity may contribute to imbalanced network activity seen in *FMR1* KO mice (Jewett et al., 2018).

The main purpose of homeostatic plasticity is to sense and regulate network excitability to a set-point value to prevent instability and optimize information processing. Studies have shown that neural network stability can be achieved in a number of ways, such as: (1) maintaining E/I balance in the network (Maffei et al., 2004; Gonzalez-Islas and Wenner, 2006; Landau et al., 2016; Keck et al., 2017); (2) regulating intrinsic neuronal firing rates in an activity-dependent manner (Desai et al., 1999; Marder and Prinz, 2003; Zhang and Linden, 2003; Joseph and Turrigiano, 2017); and (3) synaptic scaling, which up- or down-regulates excitatory synapses to modulate overall synaptic activity while maintaining the balance between synaptic weights (Turrigiano and Nelson, 2004; Davis, 2006). One of the most well-studied forms of homeostatic plasticity operating in CNS excitatory synapses is synaptic scaling. Turrigiano and colleagues were the first to demonstrate the presence of synaptic scaling in cortical neuronal culture, where they showed that tetrodotoxin (TTX)-mediated chronic blockade of neural activity caused upscaling of the strength of individual synapses. On the contrary, chronically inhibiting GABAergic transmission through the use of bicuculline or picrotoxin to promote neural activity causes a homeostatic reduction in the strength of individual excitatory synapses, with firing rates returning to baseline values following an initial elevation (Turrigiano et al., 1998). Moreover, selective activity blockade of a neuron using TTX microperfusion in its soma caused proportionate upscaling of synaptic transmission, suggesting that synaptic scaling is a cell-autonomous phenomenon (Ibata et al., 2008).

Studies investigating the signaling pathway of synaptic scaling have revealed the involvement of both N-Methyl-D-aspartate (NMDA) and α-amino-3-hydroxy-5-methyl-4-isoxazolepropionic acid (AMPA) receptors (AMPARs) in mediating homeostatic synaptic scaling at excitatory synapses (Watt et al., 2000; Maffei et al., 2004; Wierenga et al., 2005; Rodriguez et al., 2019). Synaptic upscaling in response to blocking postsynaptic transmission was shown to be achieved by increased surface expression of AMPARs and it involves the insertion of both GluA1 and GluA2 AMPAR subunits. Activity blockade in cultured neurons by TTX has been shown to enhance phosphorylation of GluA1 at the Ser845 residue. GluA1–Ser845 phosphorylation subsequently led to increased GluA1 surface accumulation in the postsynaptic compartment (Diering et al., 2014). A similar increase in GluA1–Ser845 phosphorylation was also shown to be responsible for synaptic upscaling *via* increased AMPAR–mEPSC in the visual cortex following visual deprivation (Goel et al., 2006, 2011). Apart from GluA1, the C-terminus of GluA2 alone can regulate synaptic scaling following TTX-induced synaptic upscaling *in vivo* (Gainey et al., 2009). Additionally, many other signaling molecules or postsynaptic proteins, such as brain-derived neurotrophic factor (BDNF), Arc (activity-regulated cytoskeleton-associated protein), TNFα (tumor necrosis factor α), MHC1 (major histocompatibility complex class 1), PICK1 (protein interacting with C kinase 1), β3 integrins, PSD93 (postsynaptic density protein 93), and PSD95 (postsynaptic density protein 95), also play important roles in synaptic scaling (Rutherford et al., 1998; Shepherd et al., 2006; Stellwagen and Malenka, 2006; Goddard et al., 2007; Sun and Turrigiano, 2011; Elmer and Mcallister, 2012). Compelling evidence suggests the existence of different forms of homeostatic plasticity in order to operate either as a global mechanism for all synapse types or local and specific to a certain neuronal subtype. In a nutshell, homeostatic plasticity ensures the stability of neural circuits essential for normal brain function. Because many of the genes that encode the aforementioned molecules for homeostatic plasticity are direct targets of FMRP (Niere et al., 2012; Tsai et al., 2012), it is logical to speculate that *FMR1* KO neurons may exhibit altered homeostatic plasticity. Next, we will discuss the discovery and significance of impaired homeostatic plasticity in FXS.

### Homeostatic Synaptic Plasticity Is Altered in FXS

As discussed in the previous section, loss of FMRP results in a number of changes to excitatory and inhibitory synaptic function and connectivity. There is also a plethora of studies showing that *FMR1* KO neurons fail to adjust their synaptic strength to a basal set point in response to both unconstrained network activity and activity blockade, indicative of impaired homeostatic synaptic plasticity mechanisms in FXS. In particular, there is evidence for altered regulation of AMPARs during synaptic scaling in *FMR1* KO animals (Soden and Chen, 2010; Lee et al., 2018). The surface expression of GluA1-containing AMPARs, in addition to being mediated by phosphorylation of GluA1 at the Ser845 residue as mentioned above, is also known to be regulated by the ubiquitination of multiple lysine residues in the intracellular C-terminus of GluA1 (Schwarz et al., 2010; Lin et al., 2011). Ubiquitination of GluA1 *via* the E3 ubiquitin ligase named neural precursor cell expressed developmentally down-regulated gene 4-like (Nedd4l, or Nedd4-2) leads to a reduction of surface AMPARs and this has been observed to occur during synaptic downscaling (Jewett et al., 2015). In cortical neuron cultures of *FMR1* KO mice, such Nedd4-2-mediated ubiquitination is deficient, leading to impaired synaptic downscaling (Lee et al., 2018). Mechanistically, Lee and colleagues found that dephosphorylation of Nedd4-2 following the chronic blockade of GABAergic transmission is responsible for the defect, as ectopically expressing a phospho-mimetic Nedd4-2 can restore GluA1 ubiquitination and synaptic downscaling in cultured *FMR1* KO cortical neurons.

Another reported mechanism concerning the regulation of surface AMPARs during synaptic scaling is through retinoic acid (RA) and retinoic acid receptor α (RARα) signaling in visual cortical circuits. RA regulates local homeostatic plasticity at the level of individual dendritic spines. In the case of activity blockade, a drop in Ca^2+^ levels stimulates RA synthesis, which in turn enhances local protein synthesis, increases surface insertion of GluA1-containing AMPARs, and ultimately restores Ca^2+^ levels (Aoto et al., 2008). This entire cascade of events leads to synaptic upscaling at excitatory synapses. This form of synaptic upscaling was absent in *FMR1* KO mice and could be restored by post-synaptic re-expression of FMRP (Soden and Chen, 2010). Research from the same group suggests that, apart from synaptic scaling-up at excitatory synapses (Chen et al., 2014), RA/RARα signaling also mediates inhibitory homeostatic plasticity in the mouse primary visual cortex (Zhong et al., 2018). Treatment with RA causes reduced inhibitory drive onto layer 2/3 pyramidal neurons and similar effects are triggered by visual deprivation. This RA-dependent reduction in inhibition was due to reduced inhibitory synaptic transmission from PV interneurons. Interesting, visual deprivation- and RA-dependent downregulation of inhibition was absent in the visual cortex of *FMR1* KO mice and selective deletion of *Fmr1* in PV neurons recapitulated these deficits in inhibitory synaptic downscaling. Thus, loss of FMRP in excitatory neurons impairs homeostatic up-scaling of excitatory synapses while loss of FMRP in PV inhibitory neurons impairs down-scaling of inhibitory synapses. Similar impairments in RA-dependent homeostatic plasticity were observed in *FMR1* deficient human pluripotent stem cells (Zhang et al., 2018). In addition, a recent study surprisingly revealed a physical interaction between FMRP and RARα, and such interaction mediates transcription-independent RA signaling and homeostatic plasticity (Park et al., 2021). Altogether, these findings suggest that FMRP is crucial for homeostatic synaptic plasticity, and the inability of *FMR1* deficient neurons to regulate E/I balance in the face of changes to overall activity levels may contribute to altered synaptic development and synaptic hyperexcitability in FXS.

### Homeostatic Intrinsic Plasticity Is Altered in FXS

Homeostatic synaptic plasticity is essential for preventing network hyperexcitability, particularly during early developmental periods when neuronal networks are undergoing immense modification and refinement. What other homeostatic mechanisms could be responsible for the hyperexcitability of neuronal networks in adult brains, especially in the case of FXS? One possibility lies in the homeostatic control of the intrinsic excitability of the neurons. Many studies have shown basally altered intrinsic excitability in *FMR1* KO mice, as discussed above in “Ion Channel Dysregulation and Altered Intrinsic Excitability in FXS” section. Interestingly, in line with these findings on the intrinsic properties of *FMR1* KO neurons, a recent study indicated that *FMR1* KO neurons show a significant increase in input resistance along with distinct alterations in homeostatic intrinsic plasticity in different subsets of cortical neurons. Bülow et al. (2019) found that, depending on the pattern of spikes following steps of current injections, *FMR1* KO cortical neurons exhibit strikingly different intrinsic scaling phenotypes. In comparison to WT neurons, single-spiking *FMR1* KO neurons show impaired intrinsic upscaling, whereas multispiking *FMR1* KO neurons show exaggerated intrinsic upscaling. Furthermore, Bülow and colleagues demonstrated that activity blockade in *FMR1* KO neurons alters action potential parameters, with an increase in the maximum slope of the AP rising phase (Figure 1C). This change in AP parameter in *FMR1* KO neurons may be due to increased activity of Na^+^ channels, contributing to abnormal intrinsic excitability. While the molecular mechanism underlying the differences between single-spiking and multispiking neurons during intrinsic upscaling is unclear, the study introduced the first evidence for homeostatic intrinsic plasticity deficits in *FMR1* KO mice.

Although it remains unknown how the altered homeostatic intrinsic plasticity at the single-cell level ultimately affects network stability as a whole, a recent study looking at homeostatic network plasticity may give us a clue. Jewett and colleagues demonstrated that FXS cortical neuron cultures fail to achieve homeostatic network synchronization in response to chronic activity stimulation in a multielectrode array recording (Jewett et al., 2018). This deficit was described by a novel signaling pathway, suggesting the involvement of FMRP-dependent ubiquitination of tumor suppressor p53 by the E3 ligase Mdm2 in response to chronic activity stimulation of cortical neurons. In *FMR1* KO neurons, this signaling is hampered, likely due to basally altered activity of Mdm2 (Tsai et al., 2017), and thus the homeostatic reduction in the amplitude of neuronal network spikes is absent (Jewett et al., 2018). This study, together with other studies using single-neuron recordings, suggests that the cortical neurons and networks in *FMR1* KO mice exhibit impaired homeostatic plasticity which could be responsible, at least in part, for circuit hyperexcitability and associated behavioral defects in FXS. It is likely that homeostatic plasticity disruptions are occurring in other brain areas as well. For example, Svalina and colleagues reported that principal cells in the lateral amygdala show enhanced excitability owing to reduced feed-forward inhibition, indicating a potential deficit in homeostatic plasticity in the amygdala (Svalina et al., 2021), which could be relevant to the anxiety issues in FXS.

Finally, others studies have revealed that some forms of homeostatic plasticity are intact in *FMR1* KO animals. For instance, homeostatic changes at the circuit level are normal *ex vivo* in auditory cortical slice cultures following chronic stimulation (Motanis and Buonomano, 2020). Homeostatic changes to axon initial segment (AIS) length, which plays a crucial role in neuronal excitability, are intact in CA1 hippocampal neurons of *FMR1* KO animals as well (Booker et al., 2020). However, this study also found that AIS length was increased in *FMR1* KO neurons (Figure 1B), leading to increased cellular excitability. Interestingly, these neurons had reduced functional input from the entorhinal cortex, suggesting that AIS-dependent hyperexcitability in *FMR1* KO mice may actually be an adaptive homeostatic change to compensate for reduced synaptic input. Thus, in some cases, cellular hyperexcitability observed in FX models may act to stabilize rather than destabilize circuit function, as has been suggested for changes to E/I balance in the somatosensory cortex (Antoine et al., 2019). It should be noted that decreased feed-forward inhibition (Wahlstrom-Helgren and Klyachko, 2015) and altered post-synaptic dendritic integration (Brager et al., 2012) are also observed in this entorhinal-CA1 circuit in *FMR1* KO mice, and future work must delineate the relationship between these changes to synaptic function, intrinsic properties, and AIS length. In other cases, it appears that intact homeostatic mechanisms fail to correct hyperexcitability in *FMR1* KO animals, as seen in the amygdala, where homeostatic upregulation of inhibitory synaptic transmission during critical stages of development cannot be maintained in the mature brain (Vislay et al., 2013). Therefore, while compelling evidence from the growing body of studies strongly suggests that hyperexcitability in FXS can be partially attributed to impairment in homeostatic plasticity, the contradictory results reiterate the complexity of brain hyperexcitability in FXS. More *in vivo* studies using physiological simulations would be needed to further consolidate the observations about homeostatic plasticity in FXS animal models.

## Behavioral Consequences of Hyperexcitable Circuits in FXS

The studies highlighted in the previous sections demonstrate that hyperexcitable networks are a common outcome of loss of FMRP, but that the mechanisms leading to this phenotype involve complex changes to synaptic and circuit function and plasticity that are highly region-specific. An important question is how does hyperexcitability ultimately contribute to the neurocognitive phenotypes of FXS, and how can we parse the influence of different cellular and molecular mechanisms across brain regions, as this will have important consequences for clinical treatment. The clinical features of FXS are also quite complex with multiple physical and neuropsychiatric symptoms, including intellectual disability, autistic behavior, social anxiety, perseverative behaviors, hyperactivity/impulsivity/aggression, language deficits, and disrupted sleep (Lozano et al., 2014). In a majority of cases, FX individuals exhibit sensory alterations that range from hypersensitivity to sensory stimuli and hyperarousal to seizures. These last symptoms are particularly relevant for this review, as they may provide a tractable window for understanding how hyperexcitability and homeostatic plasticity in different brain regions may relate to core behavioral impairments in FX.

### Elevated Seizure Susceptibility in FXS

Hyperexcitability has been linked to elevated susceptibility to seizures in FXS individuals. Some of the earliest works looking at epilepsy in FXS revealed that 10–20% of FXS individuals become epileptic early in childhood (Musumeci et al., 1999; Berry-Kravis, 2002). Despite an apparent epileptiform abnormality on EEG, studies suggest that the abnormal EEG pattern in FXS patients appear to resemble that of a benign focal epilepsy of childhood (BFEC; Berry-Kravis, 2002; Qiu et al., 2008) in which the patients rarely develop status epilepticus (SE; Gauthey et al., 2010). In addition, the patients usually respond well to anti-epilepsy medicine, and most of the patients enter seizure remission before adulthood (Musumeci et al., 1999; Berry-Kravis, 2002). Although seizures and epilepsy are easily controlled for most patients, these seizures are still considered one of the serious comorbidities of FXS, and the EEG pattern in FXS is used as one of the endophenotypes to guide personalized treatment (Cowley et al., 2016).

Elevated seizure susceptibility has been documented in *FMR1* KO mice as well, with the increased preponderance of audiogenic seizures (AGSs) being one of the most reliable and consistent approaches to assessing hyperexcitability *in vivo*. In AGS experiments, mice are presented with a 110–120 dB siren or alarm sound for a duration of 1–3 min. The mice are then scored for behavioral seizures with SE and death as a common final end point for the *FMR1* KO mice (Musumeci et al., 2000). Interestingly, conditional deletion of *FMR1* in subcortical glutamatergic neurons reproduces the AGS phenotype, while re-expression of FMRP selectively in the inferior colliculus of global *FMR1* KO mice eliminates AGSs (Gonzalez et al., 2019). Thus, while auditory EEG abnormalities that contribute to auditory processing deficits in *FMR1* KO mice appear to depend on altered cortical function (Goswami et al., 2019; Lovelace et al., 2020), AGSs are generated subcortically, likely within the auditory midbrain. It is also worth noting that *FMR1* KO rats did not exhibit AGSs as compared to *FMR1* KO mice (Wong et al., 2020), suggesting that AGS is likely a mouse-specific phenotype. In the model of kindling-induced seizures, despite a similar electrographic seizure threshold between *FMR1* KO mice and their WT littermates, *FMR1* KO mice displayed accelerated seizure progression both behaviorally and electrographically (Qiu et al., 2009). Despite similar susceptibility between *FMR1* KO mice and their WT littermates following systemic injections of kainic acid in the model of chemically-induced seizures, *FMR1* KO mice did not exhibit homeostatic response triggers by the seizures (Liu et al., 2021), suggesting the possibility that the *FMR1* KO mice might exhibit higher susceptibility to multiple or sequential seizures. This finding requires future investigation to validate it.

In summary, elevated seizure susceptibility is common in patients and animal models of FXS. While the seizures are usually not spontaneous, they do indicate a hyperexcitable brain circuit in FXS and provide a means for evaluating excitability imbalance in research models and testing therapeutic interventions for FXS.

### Sensory Hypersensitivity in FXS

Atypical sensory processing is a common and debilitating feature of FXS and ASD (Sinclair et al., 2017b). Sensory abnormalities are present early in development and are predictive of disease phenotypes that emerge later in life, such as increased anxiety and abnormal social behavior (Baranek et al., 2008, 2013). Sensory phenotypes in FXS can be complex, typically manifesting across sensory domains and characterized by both over- and under-responsiveness to sensory stimuli as well as avoidance and/or sensory seeking behavior (Rais et al., 2018). However, hypersensitivity to sensory stimuli is often the most common and most disruptive symptom, and this may be directly related to neuronal hyperexcitability in sensory areas. Evidence for heightened sensory sensitivity in FXS comes from validated scales and parental questionnaires, such as the Short Sensory Profile (Rogers et al., 2003; Baranek et al., 2008), as well objective measures, including increased electrodermal response to stimuli (Miller et al., 1999) and altered event-related brain potentials (ERPs; Sinclair et al., 2017b). In addition to being a clinically important aspect of the FXS phenotype, sensory dysregulation affords an opportunity to link underlying disease mechanisms to behavioral symptoms in animal models of FXS, as sensory systems are relatively well-conserved across species and there are well-characterized behavioral and electrophysiological read-outs of sensory processing.

Some of the first evidence for sensory hypersensitivity in FXS animal models came from examination of the acoustic startle response (ASR), with *FMR1* KO mice exhibiting an increase in this full body reflexive response to loud sound stimuli (Chen and Toth, 2001). More recently, the BK channel opener BMS-204352 was shown to reverse ASR increases in *FMR1* KO mice, providing a link between altered ion channel regulation, neuronal hyperexcitability, and sensory hypersensitivity (Zhang et al., 2014). However, other studies have observed a decrease (Frankland et al., 2004; Paylor et al., 2008) or no change (Mccullagh et al., 2020) in ASR in *FMR1* KO mice. The cause of these discrepancies is unclear but may be due in part to background strain effects (Errijgers et al., 2008) and methodological differences (Lauer et al., 2017). Despite the inherent variability in ASR phenotype, studies have shown that the ASR is directly related to FMRP expression (Yun et al., 2006) and ASR phenotypes in *FMR1* KO animals can be rescued with the reintroduction of the *Fmr1* gene (Paylor et al., 2008), indicating that some aspects of the ASR are directly related to loss of FMRP.

Examination of ASR in FXS humans has found no change in baseline startle responses but impaired pre-pulse inhibition of the ASR (PPI), a modification of the paradigm where a startle-eliciting sound is preceded by a lower level auditory or tactile cue that reflexively reduces ASR magnitude (Frankland et al., 2004; Hessl et al., 2009). PPI alterations are also commonly observed in *FMR1* KO mice, however often in the opposite direction as seen in humans, with enhanced rather than reduced PPI magnitude (Chen and Toth, 2001; Nielsen et al., 2002; Frankland et al., 2004; Paylor et al., 2008; Orefice et al., 2016; Kokash et al., 2019). These discrepancies may once again be due to methodological details (Hessl et al., 2009). A recent study using different PPI cues, such as gaps in sound or different spatial locations of sound sources, found decreased PPI in *FMR1* KO mice (Mccullagh et al., 2020) while no PPI alterations were observed in *FMR1* KO rats using a novel, robust methodological approach (Miller et al., 2021). Despite differences from the human phenotype, PPI alterations in *FMR1* KO animals are sensitive to treatments that also reverse auditory hyperexcitability phenotypes, such as mGluR5 inhibitors (de Vrij et al., 2008) or genetic reduction of MMP9 (Kokash et al., 2019). However, the variability in results across studies using these reflexive assays has limited their utility for understanding sensory processing issues in FX.

Operant perceptual decision-making tasks, where animals are conditioned to respond to specific stimuli, allow for quantitative assessment of sensory processing in a manner that can be directly translated to human studies. A recent study assessed sound hypersensitivity in *FMR1* KO rats using an operant sound detection task (Auerbach et al., 2021). *FMR1* KO rats learned the task at the same rate as WT counterparts and reached similar peak performance for detection of near threshold sounds. However, *FMR1* KO rats exhibited significantly faster auditory reaction times (RT) at suprathreshold intensities, suggestive of increased perceptual sensitivity. Indeed, RT-intensity functions have been shown to be a reliable psychoacoustic measure of loudness growth in both humans (Marshall and Brandt, 1980) and animal models (Radziwon and Salvi, 2020). *FMR1* KO rats also displayed abnormal integration of sound duration and bandwidth in a manner consistent with altered loudness perception. These results provide evidence for aberrant low-level auditory processing and excessive loudness growth in *FMR1* KO animals using a task design with potential for clinical translation. RT differences were also sensitive to mGluR5 inhibition, demonstrating this phenotype is related to a core molecular pathology in FXS. Future work must determine the neurophysiological correlates of this behavioral phenotype, but multiple studies have found evidence of sound-evoked hyperactivity and circuit hyperexcitability in the auditory cortex of *FMR1* KO mice (Rotschafer and Razak, 2013; Lovelace et al., 2018; Goswami et al., 2019) and FXS individuals (Van der Molen et al., 2012; Ethridge et al., 2016). In particular, increased event-related potentials (ERPs) and reduced synchronization to auditory chirp stimuli, an amplitude modulated sound that is modulated by a sinusoid with increasing or decreasing frequency, are observed in *FMR1* KO mice and FX individuals (Ethridge et al., 2017; Lovelace et al., 2018; Jonak et al., 2020). These processing deficits could underly the observed behavioral impairments in loudness perception and temporal integration.

A recent study by Goel and colleagues has provided some of the most complete evidence linking circuit hyperexcitability to sensory processing issues in FXS (Goel et al., 2018). By combining *in vivo* Ca^2+^ imaging from genetically-identified PV interneurons and putative excitatory neurons in the visual cortex of mice performing an orientation discrimination task, they found delayed perceptual learning and impaired fine-tuned discrimination in *FMR1* KO mice that correlated with deficits in orientation tuning of principal cells and reduced stimulus-evoked activity in PV neurons. Chemogenetic activation of PV neurons rescued the behavioral impairments in *FMR1* KO mice, suggesting a causal relationship between disrupted E/I balance and impaired sensory processing. Furthermore, parallel human psychophysics studies using an analogous paradigm to one used in mice found similar visual discrimination impairments in FXS individuals.

Similar hypersensitivity (He et al., 2017) and perceptual learning deficits (Arnett et al., 2014) have been observed in the tactile domain of *FMR1* KO mice. Using a novel assay for tactile defensiveness, He and colleagues found that head-fixed *FMR1* KO mice exhibited an exaggerated motor response in attempts to avoid whisker stimulation. While numerous *ex vivo* studies have found evidence for hyperexcitability in the somatosensory cortex of *FMR1* KO animals (see Section “Hyperexcitable Neurons and Networks in Fragile X Syndrome”), no differences in overall whisker-evoked activity were seen in *FMR1* KO mice in this study, as assessed by two-photon Ca^2+^ imaging of layer 2/3 neurons (He et al., 2017). This is consistent with *in vivo* cell-attached recordings showing no difference in whisker-evoked spiking activity from this same neuronal population (Antoine et al., 2019). However, He and colleagues did find a pronounced deficit in neuronal adaption to repetitive stimulation in *FMR1* KO animals, suggesting that tactile hypersensitivity may be driven in part by impaired habituation to sensory input. Similar habituation deficits have been observed in the auditory (Lovelace et al., 2016) and visual (Pak et al., 2021) domains of *FMR1* KO mice as well. Interestingly, auditory habituation measured behaviorally using ASR has been shown to depend on intact BK channel function (Typlt et al., 2013). Loss of FMRP-mediated regulation of BK channel conductance (Deng et al., 2013; Deng and Klyachko, 2016b) could therefore conceivably account for impaired habituation in *FMR1* KO animals, although this has not been directly tested yet. While less characterized, there is evidence for altered olfaction in FXS models as well (Bodaleo et al., 2019). Interestingly, studies in *FMR1* KO mice (Schilit Nitenson et al., 2015) and a FXS drosophila model (Franco et al., 2017) both found that FXS animals exhibited decreased odor sensitivity, contrary to findings from other sensory domains.

Animal model studies have highlighted several promising molecular targets for the treatment of FXS and, as discussed above, recent studies have uncovered novel treatment targets aimed at circuit-level disruptions that may work in parallel or perhaps even synergistically with existing molecular therapies. However, an important lesson learned from recent clinical trials in FXS is the need for quantitative, objective behavioral read-outs that translate between pre-clinical animal models and clinical trials (Berry-Kravis et al., 2018). Sensory processing disruptions may provide a unique behavioral platform for pre-clinical drug screening using disease-relevant phenotypes that are relatively well-conserved between humans and animal models.

## Conclusion

Here we have highlighted the number of ways in which loss of FMRP can lead to neuronal hyperexcitability, and how these cellular and circuit changes contribute to the FXS phenotype. Because FMRP regulates multiple activity-dependent processes and is regulated in an activity-dependent manner itself, it is difficult to disentangle the direct effects of FMRP loss from secondary consequences. While some of the phenotypes described above are likely to be compensatory adaptions rather than direct pathologies related to *FMR1* deletion, it is possible that both these primary and compensatory changes contribute to hyperexcitability phenotypes in FXS. The exact consequence of *FMR1* deletion at the synaptic, cellular, and circuit level also depends greatly on the brain region and developmental time point being examined. However, some general themes have emerged regarding the role of FMRP in neuronal and circuit excitability: (1) FMRP is important for activity-dependent development and refinement of synaptic connectivity and loss of FMRP during early life critical periods can lead to abnormal excitatory and inhibitory synaptic connectivity, resulting in altered E/I balance that is likely to contribute to circuit hyperexcitability; (2) FMRP is required for ongoing activity-dependent plasticity in the mature brain and seems particularly important for regulating mGluR-dependent changes to excitatory synaptic function, inhibitory transmitter release, and cellular excitability; (3) FMRP regulates the expression and function of multiple ion channels through a variety of direct and indirect mechanisms. Changes to ion channel function with loss of FMRP not only directly affect intrinsic excitability in a manner to promote hyperactivity, but can lead to profound changes in pre-synaptic release properties and post-synaptic dendritic integration, which in turn will influence synaptic function and plasticity in a variety of ways; and (4) FMRP is an important regulator of homeostatic plasticity, which is essential for stabilizing activity levels in the brain, and impairments to this stabilization mechanism are likely to contribute to circuit hyperexcitability in FXS. The wide-ranging consequences of *FMR1* deletion underscore the importance of examining multiple aspects of neuronal function (e.g., cellular excitability, synaptic plasticity, and network activity) in *FMR1* KO models under the same experimental conditions, ideally using approaches that span from single neurons to intact circuits to behavior. Future studies must also continue to make use of spatial and temporally restricted deletion of *FMR1* to parse the contribution of different cell-types, brain regions, and developmental timepoints to FXS phenotypes. The development of FMRP-tat peptides to reintroduce different FMRP segments to *FMR1* KO neurons is a powerful tool for dissociating FMRPs function *via* direct protein-protein interactions from its canonical role in translational regulation (Zhan et al., 2020; Park et al., 2021). Finally, the development of novel FXS models—such as the *FMR1* KO rat (Till et al., 2015; Golden et al., 2019; Auerbach et al., 2021) and FXS human derived iPS cells (Telias et al., 2013; Bhattacharyya and Zhao, 2016) and organoids (Kang et al., 2021), will help identify which phenotypes are most highly conserved across species and highlight new treatment strategies.

## Author Contributions

All authors contributed to the article and approved the submitted version.

## Conflict of Interest

The authors declare that the research was conducted in the absence of any commercial or financial relationships that could be construed as a potential conflict of interest.

## Publisher’s Note

All claims expressed in this article are solely those of the authors and do not necessarily represent those of their affiliated organizations, or those of the publisher, the editors and the reviewers. Any product that may be evaluated in this article, or claim that may be made by its manufacturer, is not guaranteed or endorsed by the publisher.

## References

[B1] Abbott L. F. Nelson S. B. (2000). Synaptic plasticity: taming the beast. Nat. Neurosci. 3, 1178–1183. 10.1038/81453 11127835

[B2] Abitbol M. Menini C. Delezoide A.-L. Rhyner T. Vekemans M. Mallet J. (1993). Nucleus basalis magnocellularis and hippocampus are the major sites of FMR-1 expression in the human fetal brain. Nat. Genet. 4, 147–153. 10.1038/ng0693-147 8348153

[B3] Antar L. N. Afroz R. Dictenberg J. B. Carroll R. C. Bassell G. J. (2004). Metabotropic glutamate receptor activation regulates fragile x mental retardation protein and FMR1 mRNA localization differentially in dendrites and at synapses. J. Neurosci. 24, 2648–2655. 10.1523/JNEUROSCI.0099-04.2004 15028757 PMC6729525

[B4] Antar L. N. Li C. Zhang H. Carroll R. C. Bassell G. J. (2006). Local functions for FMRP in axon growth cone motility and activity-dependent regulation of filopodia and spine synapses. Mol. Cell. Neurosci. 32, 37–48. 10.1016/j.mcn.2006.02.001 16631377

[B5] Antoine M. W. Langberg T. Schnepel P. Feldman D. E. (2019). Increased excitation-inhibition ratio stabilizes synapse and circuit excitability in four autism mouse models. Neuron 101, 648–661.e4. 10.1016/j.neuron.2018.12.026 30679017 PMC6733271

[B6] Aoto J. Nam C. I. Poon M. M. Ting P. Chen L. (2008). Synaptic signaling by all-trans retinoic acid in homeostatic synaptic plasticity. Neuron 60, 308–320. 10.1016/j.neuron.2008.08.012 18957222 PMC2634746

[B7] Arnett M. T. Herman D. H. Mcgee A. W. (2014). Deficits in tactile learning in a mouse model of fragile X syndrome. PloS One 9:e109116. 10.1371/journal.pone.0109116 25296296 PMC4189789

[B8] Ascano M. Jr Mukherjee N. Bandaru P. Miller J. B. Nusbaum J. D. Corcoran D. L.. (2012). FMRP targets distinct mRNA sequence elements to regulate protein expression. Nature 492, 382–386. 10.1038/nature11737 23235829 PMC3528815

[B9] Ashley C. T. Jr. Wilkinson K. D. Reines D. Warren S. T. (1993). FMR1 protein: conserved RNP family domains and selective RNA binding. Science 262, 563–566. 10.1126/science.7692601 7692601

[B10] Auerbach B. D. Bear M. F. (2010). Loss of the fragile X mental retardation protein decouples metabotropic glutamate receptor dependent priming of long-term potentiation from protein synthesis. J. Neurophysiol. 104, 1047–1051. 10.1152/jn.00449.2010 20554840 PMC2934918

[B11] Auerbach B. D. Manohar S. Radziwon K. Salvi R. (2021). Auditory hypersensitivity and processing deficits in a rat model of fragile X syndrome. Neurobiol. Dis. 161:105541. 10.1016/j.nbd.2021.105541 34751141

[B12] Balmer T. S. Carels V. M. Frisch J. L. Nick T. A. (2009). Modulation of perineuronal nets and parvalbumin with developmental song learning. J. Neurosci. 29, 12878–12885. 10.1523/JNEUROSCI.2974-09.2009 19828802 PMC2769505

[B13] Baranek G. T. Roberts J. E. David F. J. Sideris J. Mirrett P. L. Hatton D. D.. (2008). Developmental trajectories and correlates of sensory processing in young boys with fragile X syndrome. Phys. Occup. Ther. Pediatr. 28, 79–98. 10.1300/j006v28n01_06 18399048

[B14] Baranek G. T. Watson L. R. Boyd B. A. Poe M. D. David F. J. Mcguire L. (2013). Hyporesponsiveness to social and nonsocial sensory stimuli in children with autism, children with developmental delays and typically developing children. Dev. Psychopathol. 25, 307–320. 10.1017/S0954579412001071 23627946 PMC3641693

[B15] Bear M. F. Huber K. M. Warren S. T. (2004). The mGluR theory of fragile X mental retardation. Trends Neurosci. 27, 370–377. 10.1016/j.tins.2004.04.009 15219735

[B16] Bechara E. G. Didiot M. C. Melko M. Davidovic L. Bensaid M. Martin P.. (2009). A novel function for fragile X mental retardation protein in translational activation. PLoS Biol. 7:e16. 10.1371/journal.pbio.1000016 19166269 PMC2628407

[B17] Berry-Kravis E. (2002). Epilepsy in fragile X syndrome. Dev. Med. Child Neurol. 44, 724–728. 10.1017/s0012162201002833 12418611

[B18] Berry-Kravis E. Hagerman R. Visootsak J. Budimirovic D. Kaufmann W. E. Cherubini M.. (2017). Arbaclofen in fragile X syndrome: results of phase 3 trials. J. Neurodev. Disord. 9:3. 10.1186/s11689-016-9181-6 28616094 PMC5467054

[B19] Berry-Kravis E. M. Hessl D. Rathmell B. Zarevics P. Cherubini M. Walton-Bowen K.. (2012). Effects of STX209 (arbaclofen) on neurobehavioral function in children and adults with fragile X syndrome: a randomized, controlled, phase 2 trial. Sci. Transl. Med. 4:152ra127. 10.1126/scitranslmed.3004214 22993294

[B20] Berry-Kravis E. M. Lindemann L. Jonch A. E. Apostol G. Bear M. F. Carpenter R. L.. (2018). Drug development for neurodevelopmental disorders: lessons learned from fragile X syndrome. Nat. Rev. Drug Discov. 17, 280–299. 10.1038/nrd.2017.221 29217836 PMC6904225

[B21] Beston B. R. Jones D. G. Murphy K. M. (2010). Experience-dependent changes in excitatory and inhibitory receptor subunit expression in visual cortex. Front. Synaptic Neurosci. 2:138. 10.3389/fnsyn.2010.00138 21423524 PMC3059668

[B22] Bhakar A. L. Dolen G. Bear M. F. (2012). The pathophysiology of fragile X (and what it teaches us about synapses). Annu. Rev. Neurosci. 35, 417–443. 10.1146/annurev-neuro-060909-153138 22483044 PMC4327822

[B23] Bhattacharyya A. Zhao X. (2016). Human pluripotent stem cell models of Fragile X Syndrome. Mol. Cell. Neurosci. 73, 43–51. 10.1016/j.mcn.2015.11.011 26640241 PMC4867245

[B24] Bhogal B. Jongens T. A. (2010). Fragile X syndrome and model organisms: identifying potential routes of therapeutic intervention. Dis. Model. Mech. 3, 693–700. 10.1242/dmm.002006 20682752 PMC2965397

[B25] Bianchi R. Chuang S. C. Zhao W. Young S. R. Wong R. K. (2009). Cellular plasticity for group I mGluR-mediated epileptogenesis. J. Neurosci. 29, 3497–3507. 10.1523/JNEUROSCI.5447-08.2009 19295155 PMC2692254

[B26] Bilousova T. V. Dansie L. Ngo M. Aye J. Charles J. R. Ethell D. W.. (2009). Minocycline promotes dendritic spine maturation and improves behavioural performance in the fragile X mouse model. J. Med. Genet. 46, 94–102. 10.1136/jmg.2008.061796 18835858

[B27] Bodaleo F. Tapia-Monsalves C. Cea-Del Rio C. Gonzalez-Billault C. Nunez-Parra A. (2019). Structural and functional abnormalities in the olfactory system of fragile X syndrome models. Front. Mol. Neurosci. 12:135. 10.3389/fnmol.2019.00135 31191246 PMC6548058

[B28] Bonaccorso C. M. Spatuzza M. Di Marco B. Gloria A. Barrancotto G. Cupo A.. (2015). Fragile X mental retardation protein (FMRP) interacting proteins exhibit different expression patterns during development. Int. J. Dev. Neurosci. 42, 15–23. 10.1016/j.ijdevneu.2015.02.004 25681562

[B29] Booker S. A. Domanski A. P. F. Dando O. R. Jackson A. D. Isaac J. T. R. Hardingham G. E.. (2019). Altered dendritic spine function and integration in a mouse model of fragile X syndrome. Nat. Commun. 10:4813. 10.1038/s41467-019-11891-6 31645626 PMC6811549

[B30] Booker S. A. Simoes De Oliveira L. Anstey N. J. Kozic Z. Dando O. R. Jackson A. D.. (2020). Input-output relationship of CA1 pyramidal neurons reveals intact homeostatic mechanisms in a mouse model of fragile X syndrome. Cell Rep. 32:107988. 10.1016/j.celrep.2020.107988 32783927 PMC7435362

[B31] Bourgeron T. (2015). From the genetic architecture to synaptic plasticity in autism spectrum disorder. Nat. Rev. Neurosci. 16, 551–563. 10.1038/nrn3992 26289574

[B32] Brager D. H. Akhavan A. R. Johnston D. (2012). Impaired dendritic expression and plasticity of h-channels in the fmr1(-/y) mouse model of fragile X syndrome. Cell Rep. 1, 225–233. 10.1016/j.celrep.2012.02.002 22662315 PMC3363364

[B33] Brandalise F. Kalmbach B. E. Mehta P. Thornton O. Johnston D. Zemelman B. V.. (2020). Fragile X mental retardation protein bidirectionally controls dendritic Ih in a cell type-specific manner between mouse hippocampus and prefrontal cortex. J. Neurosci. 40, 5327–5340. 10.1523/JNEUROSCI.1670-19.2020 32467357 PMC7329306

[B35] Brown V. Jin P. Ceman S. Darnell J. C. O’donnell W. T. Tenenbaum S. A.. (2001). Microarray identification of FMRP-associated brain mRNAs and altered mRNA translational profiles in fragile X syndrome. Cell 107, 477–487. 10.1016/s0092-8674(01)00568-2 11719188

[B34] Brown M. R. Kronengold J. Gazula V.-R. Chen Y. Strumbos J. G. Sigworth F. J.. (2010). Fragile X mental retardation protein controls gating of the sodium-activated potassium channel Slack. Nat. Neurosci. 13, 819–821. 10.1038/nn.2563 20512134 PMC2893252

[B2880] Budimirovic D. B. Dominick K. C. Gabis L. V. Adams M. Adera M. Huang L.. (2021). Gaboxadol in Fragile X syndrome: a 12-week randomized, double-blind, parallel-group, phase 2a study. Front. Pharmacol. 12:757825. 10.3389/fphar.2021.757825 34690787 PMC8531725

[B36] Bülow P. Murphy T. J. Bassell G. J. Wenner P. (2019). Homeostatic intrinsic plasticity is functionally altered in Fmr1 KO cortical neurons. Cell Rep. 26, 1378–1388.e3. 10.1016/j.celrep.2019.01.035 30726724 PMC6443253

[B37] Bureau I. Shepherd G. M. Svoboda K. (2008). Circuit and plasticity defects in the developing somatosensory cortex of FMR1 knock-out mice. J. Neurosci. 28, 5178–5188. 10.1523/JNEUROSCI.1076-08.2008 18480274 PMC2696604

[B38] Cardin J. A. Carlen M. Meletis K. Knoblich U. Zhang F. Deisseroth K.. (2009). Driving fast-spiking cells induces gamma rhythm and controls sensory responses. Nature 459, 663–667. 10.1038/nature08002 19396156 PMC3655711

[B39] Carreno-Munoz M. I. Martins F. Medrano M. C. Aloisi E. Pietropaolo S. Dechaud C.. (2018). Potential involvement of impaired BKCa channel function in sensory defensiveness and some behavioral disturbances induced by unfamiliar environment in a mouse model of fragile X syndrome. Neuropsychopharmacology 43, 492–502. 10.1038/npp.2017.149 28722023 PMC5770751

[B40] Ceman S. O’donnell W. T. Reed M. Patton S. Pohl J. Warren S. T. (2003). Phosphorylation influences the translation state of FMRP-associated polyribosomes. Hum. Mol. Genet. 12, 3295–3305. 10.1093/hmg/ddg350 14570712

[B41] Centonze D. Rossi S. Mercaldo V. Napoli I. Ciotti M. T. De Chiara V.. (2008). Abnormal striatal GABA transmission in the mouse model for the fragile X syndrome. Biol. Psychiatry 63, 963–973. 10.1016/j.biopsych.2007.09.008 18028882

[B45] Chen R. Cros D. Curra A. Di Lazzaro V. Lefaucheur J. P. Magistris M. R.. (2008). The clinical diagnostic utility of transcranial magnetic stimulation: report of an IFCN committee. Clin. Neurophysiol. 119, 504–532. 10.1016/j.clinph.2007.10.014 18063409

[B42] Chen L. Lau A. G. Sarti F. (2014). Synaptic retinoic acid signaling and homeostatic synaptic plasticity. Neuropharmacology 78, 3–12. 10.1016/j.neuropharm.2012.12.004 23270606 PMC3884035

[B43] Chen L. Toth M. (2001). Fragile X mice develop sensory hyperreactivity to auditory stimuli. Neuroscience 103, 1043–1050. 10.1016/s0306-4522(01)00036-7 11301211

[B44] Chen L. Yun S. W. Seto J. Liu W. Toth M. (2003). The fragile x mental retardation protein binds and regulates a novel class of mRNAs containing u rich target sequences. Neuroscience 120, 1005–1017. 10.1016/s0306-4522(03)00406-8 12927206

[B46] Chen X. Yuan L.-L. Zhao C. Birnbaum S. G. Frick A. Jung W. E.. (2006). Deletion of Kv4.2 gene eliminates dendritic A-type K+ current and enhances induction of long-term potentiation in hippocampal CA1 pyramidal neurons. J. Neurosci. 26, 12143–12151. 10.1523/JNEUROSCI.2667-06.2006 17122039 PMC6675426

[B47] Christie S. B. Akins M. R. Schwob J. E. Fallon J. R. (2009). The FXG: a presynaptic fragile X granule expressed in a subset of developing brain circuits. J. Neurosci. 29, 1514–1524. 10.1523/JNEUROSCI.3937-08.2009 19193898 PMC2746438

[B48] Chuang S. C. Zhao W. Bauchwitz R. Yan Q. Bianchi R. Wong R. K. (2005). Prolonged epileptiform discharges induced by altered group I metabotropic glutamate receptor-mediated synaptic responses in hippocampal slices of a fragile X mouse model. J. Neurosci. 25, 8048–8055. 10.1523/JNEUROSCI.1777-05.2005 16135762 PMC6725444

[B49] Collingridge G. L. Peineau S. Howland J. G. Wang Y. T. (2010). Long-term depression in the CNS. Nat. Rev. Neurosci. 11, 459–473. 10.1038/nrn2867 20559335

[B50] Comery T. A. Harris J. B. Willems P. J. Oostra B. A. Irwin S. A. Weiler I. J.. (1997). Abnormal dendritic spines in fragile X knockout mice: maturation and pruning deficits. Proc. Natl. Acad. Sci. U S A 94, 5401–5404. 10.1073/pnas.94.10.5401 9144249 PMC24690

[B51] Contractor A. Klyachko V. A. Portera-Cailliau C. (2015). Altered neuronal and circuit excitability in fragile X syndrome. Neuron 87, 699–715. 10.1016/j.neuron.2015.06.017 26291156 PMC4545495

[B52] Cowley B. Kirjanen S. Partanen J. Castrén M. L. (2016). Epileptic electroencephalography profile associates with attention problems in children with fragile X syndrome: review and case series. Front. Hum. Neurosci. 10:353. 10.3389/fnhum.2016.00353 27462212 PMC4941803

[B53] Cruz-Martín A. Crespo M. Portera-Cailliau C. (2010). Delayed stabilization of dendritic spines in fragile X mice. J. Neurosci. 30, 7793–7803. 10.1523/JNEUROSCI.0577-10.2010 20534828 PMC2903441

[B54] Curia G. Papouin T. Seguela P. Avoli M. (2009). Downregulation of tonic GABAergic inhibition in a mouse model of fragile X syndrome. Cereb. Cortex 19, 1515–1520. 10.1093/cercor/bhn159 18787232 PMC4873279

[B55] D’hulst C. De Geest N. Reeve S. P. Van Dam D. De Deyn P. P. Hassan B. A.. (2006). Decreased expression of the GABAA receptor in fragile X syndrome. Brain Res. 1121, 238–245. 10.1016/j.brainres.2006.08.115 17046729

[B56] D’hulst C. Heulens I. Aa N. V. D. Goffin K. Koole M. Porke K.. (2015). Positron emission tomography (pet) quantification of GABAA receptors in the brain of fragile X patients. PLoS One 10:e0131486. 10.1371/journal.pone.0131486 26222316 PMC4519313

[B57] Danesi C. Achuta V. S. Corcoran P. Peteri U. K. Turconi G. Matsui N.. (2018). Increased calcium influx through L-type calcium channels in human and mouse neural progenitors lacking fragile X mental retardation protein. Stem Cell Rep. 11, 1449–1461. 10.1016/j.stemcr.2018.11.003 30503263 PMC6294261

[B58] Darnell J. C. Van Driesche S. J. Zhang C. Hung K. Y. Mele A. Fraser C. E.. (2011). FMRP stalls ribosomal translocation on mRNAs linked to synaptic function and autism. Cell 146, 247–261. 10.1016/j.cell.2011.06.013 21784246 PMC3232425

[B59] Davis G. W. (2006). Homeostatic control of neural activity: from phenomenology to molecular design. Ann. Rev. Neurosci. 29, 307–323. 10.1146/annurev.neuro.28.061604.135751 16776588

[B60] de Vrij F. M. S. Levenga J. Van Der Linde H. C. Koekkoek S. K. De Zeeuw C. I. Nelson D. L.. (2008). Rescue of behavioral phenotype and neuronal protrusion morphology in Fmr1 KO mice. Neurobiol. Dis. 31, 127–132. 10.1016/j.nbd.2008.04.002 18571098 PMC2481236

[B61] Defelipe J. López-Cruz P. L. Benavides-Piccione R. Bielza C. Larrañaga P. Anderson S.. (2013). New insights into the classification and nomenclature of cortical GABAergic interneurons. Nat. Rev. Neurosci. 14, 202–216. 10.1038/nrn3444 23385869 PMC3619199

[B62] Deng P.-Y. Carlin D. Oh Y. M. Myrick L. K. Warren S. T. Cavalli V.. (2019). Voltage-independent SK-channel dysfunction causes neuronal hyperexcitability in the hippocampus of Fmr1 knock-out mice. J. Neurosci. 39, 28–43. 10.1523/JNEUROSCI.1593-18.2018 30389838 PMC6325266

[B63] Deng P.-Y. Klyachko V. A. (2016a). Increased persistent sodium current causes neuronal hyperexcitability in the entorhinal cortex of Fmr1 knockout mice. Cell Rep. 16, 3157–3166. 10.1016/j.celrep.2016.08.046 27653682 PMC5055130

[B66] Deng P.-Y. Klyachko V. A. (2016b). Genetic upregulation of BK channel activity normalizes multiple synaptic and circuit defects in a mouse model of fragile X syndrome. J. Physiol. 594, 83–97. 10.1113/JP271031 26427907 PMC4704506

[B64] Deng P.-Y. Klyachko V. A. (2021). Channelopathies in fragile X syndrome. Nat. Rev. Neurosci. 22, 275–289. 10.1038/s41583-021-00445-9 33828309 PMC8863066

[B65] Deng P.-Y. Rotman Z. Blundon J. A. Cho Y. Cui J. Cavalli V.. (2013). FMRP regulates neurotransmitter release and synaptic information transmission by modulating action potential duration *via* BK channels. Neuron 77, 696–711. 10.1016/j.neuron.2012.12.018 23439122 PMC3584349

[B68] Desai N. S. Rutherford L. C. Turrigiano G. G. (1999). Plasticity in the intrinsic excitability of cortical pyramidal neurons. Nat. Neurosci. 2, 515–520. 10.1038/9165 10448215

[B69] Devys D. Lutz Y. Rouyer N. Bellocq J. P. Mandel J. L. (1993). The FMR-1 protein is cytoplasmic, most abundant in neurons and appears normal in carriers of a fragile X premutation. Nat. Genet. 4, 335–340. 10.1038/ng0893-335 8401578

[B70] Diering G. H. Gustina A. S. Huganir R. L. (2014). PKA-GluA1 coupling *via* AKAP5 controls AMPA receptor phosphorylation and cell-surface targeting during bidirectional homeostatic plasticity. Neuron 84, 790–805. 10.1016/j.neuron.2014.09.024 25451194 PMC4254581

[B71] Domanski A. P. F. Booker S. A. Wyllie D. J. A. Isaac J. T. R. Kind P. C. (2019). Cellular and synaptic phenotypes lead to disrupted information processing in Fmr1–KO mouse layer 4 barrel cortex. Nat. Commun. 10:4814. 10.1038/s41467-019-12736-y 31645553 PMC6811545

[B72] El-Hassar L. Song L. Tan W. J. T. Large C. H. Alvaro G. Santos-Sacchi J.. (2019). Modulators of Kv3 Potassium channels rescue the auditory function of fragile X mice. J. Neurosci. 39, 4797–4813. 10.1523/JNEUROSCI.0839-18.2019 30936239 PMC6561694

[B73] El Idrissi A. Ding X.-H. Scalia J. Trenkner E. Brown W. T. Dobkin C. (2005). Decreased GABA(A) receptor expression in the seizure-prone fragile X mouse. Neurosci. Lett. 377, 141–146. 10.1016/j.neulet.2004.11.087 15755515

[B74] Elmer B. M. Mcallister A. K. (2012). Major histocompatibility complex class I proteins in brain development and plasticity. Trends Neurosci. 35, 660–770. 10.1016/j.tins.2012.08.001 22939644 PMC3493469

[B75] Errijgers V. Fransen E. D’hooge R. De Deyn P. P. Kooy R. F. (2008). Effect of genetic background on acoustic startle response in fragile X knockout mice. Genet. Res. (Camb) 90, 341–345. 10.1017/S0016672308009415 18840308

[B76] Ethridge L. E. White S. P. Mosconi M. W. Wang J. Byerly M. J. Sweeney J. A. (2016). Reduced habituation of auditory evoked potentials indicate cortical hyper-excitability in Fragile X Syndrome. Transl. Psychiatry 6:e787. 10.1038/tp.2016.48 27093069 PMC4872406

[B77] Ethridge L. E. White S. P. Mosconi M. W. Wang J. Pedapati E. V. Erickson C. A.. (2017). Neural synchronization deficits linked to cortical hyper-excitability and auditory hypersensitivity in fragile X syndrome. Mol. Autism 8:22. 10.1186/s13229-017-0140-1 28596820 PMC5463459

[B78] Fahling M. Mrowka R. Steege A. Kirschner K. M. Benko E. Forstera B.. (2009). Translational regulation of the human achaete-scute homologue-1 by fragile X mental retardation protein. J. Biol. Chem. 284, 4255–4266. 10.1074/jbc.M807354200 19097999

[B79] Farrant M. Nusser Z. (2005). Variations on an inhibitory theme: phasic and tonic activation of GABAA receptors. Nat. Rev. Neurosci. 6, 215–229. 10.1038/nrn1625 15738957

[B80] Faust T. E. Gunner G. Schafer D. P. (2021). Mechanisms governing activity-dependent synaptic pruning in the developing mammalian CNS. Nat. Rev. Neurosci. 22, 657–673. 10.1038/s41583-021-00507-y 34545240 PMC8541743

[B81] Feng Y. Absher D. Eberhart D. E. Brown V. Malter H. E. Warren S. T. (1997). FMRP associates with polyribosomes as an mRNP and the I304N mutation of severe fragile X syndrome abolishes this association. Mol. Cell 1, 109–118. 10.1016/s1097-2765(00)80012-x 9659908

[B82] Ferron L. Nieto-Rostro M. Cassidy J. S. Dolphin A. C. (2014). Fragile X mental retardation protein controls synaptic vesicle exocytosis by modulating N-type calcium channel density. Nat. Commun. 5:3628. 10.1038/ncomms4628 24709664 PMC3982139

[B83] Franco L. M. Okray Z. Linneweber G. A. Hassan B. A. Yaksi E. (2017). Reduced lateral inhibition impairs olfactory computations and behaviors in a drosophila model of fragile X syndrome. Curr. Biol. 27, 1111–1123. 10.1016/j.cub.2017.02.065 28366741 PMC5405172

[B84] Frankland P. W. Wang Y. Rosner B. Shimizu T. Balleine B. W. Dykens E. M.. (2004). Sensorimotor gating abnormalities in young males with fragile X syndrome and Fmr1-knockout mice. Mol. Psychiatry 9, 417–425. 10.1038/sj.mp.4001432 14981523

[B85] Frere S. Slutsky I. (2018). Alzheimer’s disease: from firing instability to homeostasis network collapse. Neuron 97, 32–58. 10.1016/j.neuron.2017.11.028 29301104

[B86] Gainey M. A. Hurvitz-Wolff J. R. Lambo M. E. Turrigiano G. G. (2009). Synaptic scaling requires the GluR2 subunit of the AMPA receptor. J. Neurosci. 29, 6479–6489. 10.1523/JNEUROSCI.3753-08.2009 19458219 PMC2714274

[B87] Galvez R. Greenough W. T. (2005). Sequence of abnormal dendritic spine development in primary somatosensory cortex of a mouse model of the fragile X mental retardation syndrome. Am. J. Med. Genet. A 135, 155–160. 10.1002/ajmg.a.30709 15880753

[B88] Gantois I. Vandesompele J. Speleman F. Reyniers E. D’hooge R. Severijnen L.-A.. (2006). Expression profiling suggests underexpression of the GABA(A) receptor subunit delta in the fragile X knockout mouse model. Neurobiol. Dis. 21, 346–357. 10.1016/j.nbd.2005.07.017 16199166

[B89] Garcia-Pino E. Gessele N. Koch U. (2017). Enhanced excitatory connectivity and disturbed sound processing in the auditory brainstem of fragile X mice. J. Neurosci. 37, 7403–7419. 10.1523/JNEUROSCI.2310-16.2017 28674175 PMC6596706

[B90] Gatto C. L. Pereira D. Broadie K. (2014). GABAergic circuit dysfunction in the Drosophila Fragile X syndrome model. Neurobiol. Dis. 65, 142–159. 10.1016/j.nbd.2014.01.008 24423648 PMC3988906

[B91] Gauthey M. Poloni C. B. Ramelli G. P. Roulet-Perez E. Korff C. M. (2010). Status epilepticus in fragile X syndrome. Epilepsia 51, 2470–2473. 10.1111/j.1528-1167.2010.02761.x 21204809

[B92] Gholizadeh S. Halder S. K. Hampson D. R. (2015). Expression of fragile X mental retardation protein in neurons and glia of the developing and adult mouse brain. Brain Res. 1596, 22–30. 10.1016/j.brainres.2014.11.023 25446451

[B93] Gibson J. R. Bartley A. F. Hays S. A. Huber K. M. (2008). Imbalance of neocortical excitation and inhibition and altered UP states reflect network hyperexcitability in the mouse model of fragile X syndrome. J. Neurophysiol. 100, 2615–2626. 10.1152/jn.90752.2008 18784272 PMC2585391

[B94] Gladding C. M. Fitzjohn S. M. Molnar E. (2009). Metabotropic glutamate receptor-mediated long-term depression: molecular mechanisms. Pharmacol. Rev. 61, 395–412. 10.1124/pr.109.001735 19926678 PMC2802426

[B95] Goddard C. A. Butts D. A. Shatz C. J. (2007). Regulation of CNS synapses by neuronal MHC class I. Proc. Natl. Acad. Sci. U S A 104, 6828–6833. 10.1073/pnas.0702023104 17420446 PMC1871870

[B96] Godfraind J. M. Reyniers E. De Boulle K. D’hooge R. De Deyn P. P. Bakker C. E.. (1996). Long-term potentiation in the hippocampus of fragile X knockout mice. Am. J. Med. Genet. 64, 246–251. 10.1002/(SICI)1096-8628(19960809)64:2<246::AID-AJMG2>3.0.CO;2-S 8844057

[B97] Goel A. Cantu D. A. Guilfoyle J. Chaudhari G. R. Newadkar A. Todisco B.. (2018). Impaired perceptual learning in a mouse model of Fragile X syndrome is mediated by parvalbumin neuron dysfunction and is reversible. Nat. Neurosci. 21, 1404–1411. 10.1038/s41593-018-0231-0 30250263 PMC6161491

[B98] Goel A. Jiang B. Xu L. W. Song L. Kirkwood A. Lee H. K. (2006). Cross-modal regulation of synaptic AMPA receptors in primary sensory cortices by visual experience. Nat. Neurosci. 9, 1001–1003. 10.1038/nn1725 16819524 PMC1905492

[B99] Goel A. Xu L. W. Snyder K. P. Song L. Goenaga-Vazquez Y. Megill A.. (2011). Phosphorylation of ampa receptors is required for sensory deprivation-induced homeostatic synaptic plasticity. PLoS One 6:e18264. 10.1371/journal.pone.0018264 21483826 PMC3069067

[B100] Golden C. E. M. Breen M. S. Koro L. Sonar S. Niblo K. Browne A.. (2019). Deletion of the KH1 domain of Fmr1 leads to transcriptional alterations and attentional deficits in rats. Cereb. Cortex 29, 2228–2244. 10.1093/cercor/bhz029 30877790 PMC6458915

[B101] Goncalves J. T. Anstey J. E. Golshani P. Portera-Cailliau C. (2013). Circuit level defects in the developing neocortex of Fragile X mice. Nat. Neurosci. 16, 903–909. 10.1038/nn.3415 23727819 PMC3695061

[B103] Gonzalez D. Tomasek M. Hays S. Sridhar V. Ammanuel S. Chang C. W.. (2019). Audiogenic seizures in the Fmr1 knock-out mouse are induced by Fmr1 deletion in subcortical, VGlut2-expressing excitatory neurons and require deletion in the inferior colliculus. J. Neurosci. 39, 9852–9863. 10.1523/JNEUROSCI.0886-19.2019 31666356 PMC6891051

[B102] Gonzalez-Islas C. Wenner P. (2006). Spontaneous network activity in the embryonic spinal cord regulates AMPAergic and GABAergic synaptic strength. Neuron 49, 563–575. 10.1016/j.neuron.2006.01.017 16476665

[B104] Goswami S. Cavalier S. Sridhar V. Huber K. M. Gibson J. R. (2019). Local cortical circuit correlates of altered EEG in the mouse model of Fragile X syndrome. Neurobiol. Dis. 124, 563–572. 10.1016/j.nbd.2019.01.002 30639292 PMC6371815

[B105] Gross C. Yao X. Pong D. L. Jeromin A. Bassell G. J. (2011). Fragile X mental retardation protein regulates protein expression and mrna translation of the potassium channel Kv4.2. J. Neurosci. 31, 5693–5698. 10.1523/JNEUROSCI.6661-10.2011 21490210 PMC3089949

[B106] Grothe B. Pecka M. Mcalpine D. (2010). Mechanisms of sound localization in mammals. Physiol. Rev. 90, 983–1012. 10.1152/physrev.00026.2009 20664077

[B107] Guo W. Molinaro G. Collins K. A. Hays S. A. Paylor R. Worley P. F.. (2016). Selective disruption of metabotropic glutamate receptor 5-homer interactions mimics phenotypes of fragile X syndrome in mice. J. Neurosci. 36, 2131–2147. 10.1523/JNEUROSCI.2921-15.2016 26888925 PMC4756152

[B108] Hagerman R. J. Berry-Kravis E. Hazlett H. C. Bailey D. B. Moine H. Kooy R. F.. (2017). Fragile X syndrome. Nat. Rev. Dis. Primers 3:17065. 10.1038/nrdp.2017.65 28960184

[B109] Haider B. Duque A. Hasenstaub A. R. Mccormick D. A. (2006). Neocortical network activity *in vivo* is generated through a dynamic balance of excitation and inhibition. J. Neurosci. 26, 4535–4545. 10.1523/JNEUROSCI.5297-05.2006 16641233 PMC6674060

[B110] Hammond L. S. Macias M. M. Tarleton J. C. Shashidhar Pai G. (1997). Fragile X syndrome and deletions in FMR1: new case and review of the literature. Am. J. Med. Genet. 72, 430–434. 9375726

[B111] Hanson J. E. Madison D. V. (2007). Presynaptic FMR1 genotype influences the degree of synaptic connectivity in a mosaic mouse model of fragile X syndrome. J. Neurosci. 27, 4014–4018. 10.1523/JNEUROSCI.4717-06.2007 17428978 PMC6672544

[B112] Harlow E. G. Till S. M. Russell T. A. Wijetunge L. S. Kind P. Contractor A. (2010). Critical period plasticity is disrupted in the barrel cortex of Fmr1 knockout mice. Neuron 65, 385–398. 10.1016/j.neuron.2010.01.024 20159451 PMC2825250

[B113] Hays S. A. Huber K. M. Gibson J. R. (2011). Altered neocortical rhythmic activity states in Fmr1 KO mice are due to enhanced mGluR5 signaling and involve changes in excitatory circuitry. J. Neurosci. 31, 14223–14234. 10.1523/JNEUROSCI.3157-11.2011 21976507 PMC3207280

[B116] He Q. Arroyo E. D. Smukowski S. N. Xu J. Piochon C. Savas J. N.. (2018). Critical period inhibition of NKCC1 rectifies synapse plasticity in the somatosensory cortex and restores adult tactile response maps in fragile X mice. Mol. Psychiatry 24, 1732–1747. 10.1038/s41380-018-0048-y 29703945 PMC6204122

[B114] He C. X. Cantu D. A. Mantri S. S. Zeiger W. A. Goel A. Portera-Cailliau C. (2017). Tactile defensiveness and impaired adaptation of neuronal activity in the Fmr1 knock-out mouse model of autism. J. Neurosci. 37, 6475–6487. 10.1523/JNEUROSCI.0651-17.2017 28607173 PMC5511879

[B117] He Q. Nomura T. Xu J. Contractor A. (2014). The developmental switch in GABA polarity is delayed in fragile X mice. J. Neurosci. 34, 446–450. 10.1523/JNEUROSCI.4447-13.2014 24403144 PMC6608154

[B115] He C. X. Portera-Cailliau C. (2013). The trouble with spines in fragile X syndrome: density, maturity and plasticity. Neuroscience 251, 120–128. 10.1016/j.neuroscience.2012.03.049 22522472 PMC3422423

[B118] Henderson C. Wijetunge L. Kinoshita M. N. Shumway M. Hammond R. S. Postma F. R.. (2012). Reversal of disease-related pathologies in the fragile X mouse model by selective activation of GABAB receptors with arbaclofen. Sci. Transl. Med. 4:152ra128. 10.1126/scitranslmed.3004218 22993295 PMC8826584

[B119] Hessl D. R. Berry-Kravis E. Cordeiro L. Yuhas J. Ornitz E. M. Campbell A.. (2009). Prepulse inhibition in fragile X syndrome: feasibility, reliability and implications for treatment. Am. J. Med. Genet. B. Neuropsychiatr. Genet. 150B, 545–553. 10.1002/ajmg.b.30858 18785205 PMC2693303

[B120] Hinton V. J. Brown W. T. Wisniewski K. Rudelli R. D. (1991). Analysis of neocortex in three males with the fragile X syndrome. Am. J. Med. Genet. 41, 289–294. 10.1002/ajmg.1320410306 1724112

[B121] Hou L. Antion M. D. Hu D. Spencer C. M. Paylor R. Klann E. (2006). Dynamic translational and proteasomal regulation of fragile X mental retardation protein controls mGluR-dependent long-term depression. Neuron 51, 441–454. 10.1016/j.neuron.2006.07.005 16908410

[B122] Hu H. Qin Y. Bochorishvili G. Zhu Y. Van Aelst L. Zhu J. J. (2008). Ras signaling mechanisms underlying impaired GluR1-dependent plasticity associated with fragile X syndrome. J. Neurosci. 28, 7847–7862. 10.1523/JNEUROSCI.1496-08.2008 18667617 PMC2553221

[B123] Huber K. M. Gallagher S. M. Warren S. T. Bear M. F. (2002). Altered synaptic plasticity in a mouse model of fragile X mental retardation. Proc. Natl. Acad. Sci. U S A 99, 7746–7750. 10.1073/pnas.122205699 12032354 PMC124340

[B124] Huber K. M. Kayser M. S. Bear M. F. (2000). Role for rapid dendritic protein synthesis in hippocampal mGluR-dependent long-term depression. Science 288, 1254–1257. 10.1126/science.288.5469.1254 10818003

[B125] Ibata K. Sun Q. Turrigiano G. G. (2008). Rapid synaptic scaling induced by changes in postsynaptic firing. Neuron 57, 819–826. 10.1016/j.neuron.2008.02.031 18367083

[B126] Irwin S. A. Patel B. Idupulapati M. Harris J. B. Crisostomo R. A. Larsen B. P.. (2001). Abnormal dendritic spine characteristics in the temporal and visual cortices of patients with fragile-X syndrome: a quantitative examination. Am. J. Med. Genet. 98, 161–167. 10.1002/1096-8628(20010115)98:2<161::aid-ajmg1025>3.0.co;2-b 11223852

[B127] Jacquemont S. Pacini L. Jønch A. E. Cencelli G. Rozenberg I. He Y.. (2018). Protein synthesis levels are increased in a subset of individuals with fragile X syndrome. Hum. Mol. Genet. 27, 2039–2051. 10.1093/hmg/ddy099 29590342 PMC5985734

[B128] Janusz A. Milek J. Perycz M. Pacini L. Bagni C. Kaczmarek L.. (2013). The Fragile X mental retardation protein regulates matrix metalloproteinase 9 mRNA at synapses. J. Neurosci. 33, 18234–18241. 10.1523/JNEUROSCI.2207-13.2013 24227732 PMC6619756

[B129] Jewett K. A. Lee K. Y. Eagleman D. E. Soriano S. Tsai N. P. (2018). Dysregulation and restoration of homeostatic network plasticity in fragile X syndrome mice. Neuropharmacology 138, 182–192. 10.1016/j.neuropharm.2018.06.011 29890190 PMC6349417

[B130] Jewett K. A. Zhu J. Tsai N. P. (2015). The tumor suppressor p53 guides GluA1 homeostasis through Nedd4–2 during chronic elevation of neuronal activity. J. Neurochem. 135, 226–233. 10.1111/jnc.13271 26250624

[B131] Jonak C. R. Lovelace J. W. Ethell I. M. Razak K. A. Binder D. K. (2020). Multielectrode array analysis of EEG biomarkers in a mouse model of Fragile X Syndrome. Neurobiol. Dis. 138:104794. 10.1016/j.nbd.2020.104794 32036032 PMC9038039

[B132] Joseph A. Turrigiano G. G. (2017). All for one but not one for all: Excitatory synaptic scaling and intrinsic excitability are coregulated by CaMKIV, whereas inhibitory synaptic scaling is under independent control. J. Neurosci. 37, 6778–6785. 10.1523/JNEUROSCI.0618-17.2017 28592691 PMC5508258

[B133] Kang Y. Zhou Y. Li Y. Han Y. Xu J. Niu W.. (2021). A human forebrain organoid model of fragile X syndrome exhibits altered neurogenesis and highlights new treatment strategies. Nat. Neurosci. 24, 1377–1391. 10.1038/s41593-021-00913-6 34413513 PMC8484073

[B134] Kavalali E. T. Monteggia L. M. (2020). Targeting homeostatic synaptic plasticity for treatment of mood disorders. Neuron 106, 715–726. 10.1016/j.neuron.2020.05.015 32497508 PMC7517590

[B135] Keck T. Hübener M. Bonhoeffer T. (2017). Interactions between synaptic homeostatic mechanisms: an attempt to reconcile BCM theory, synaptic scaling and changing excitation/inhibition balance. Curr. Opin. Neurobiol. 43, 87–93. 10.1016/j.conb.2017.02.003 28236778

[B136] Kennedy T. Rinker D. Broadie K. (2020). Genetic background mutations drive neural circuit hyperconnectivity in a fragile X syndrome model. BMC Biol. 18:94. 10.1186/s12915-020-00817-0 32731855 PMC7392683

[B137] Kim H. Gibboni R. Kirkhart C. Bao S. (2013). Impaired critical period plasticity in primary auditory cortex of fragile X model mice. J. Neurosci. 33, 15686–15692. 10.1523/JNEUROSCI.3246-12.2013 24089476 PMC3787494

[B139] Koekkoek S. K. Yamaguchi K. Milojkovic B. A. Dortland B. R. Ruigrok T. J. Maex R.. (2005). Deletion of FMR1 in Purkinje cells enhances parallel fiber LTD, enlarges spines and attenuates cerebellar eyelid conditioning in Fragile X syndrome. Neuron 47, 339–352. 10.1016/j.neuron.2005.07.005 16055059

[B140] Koga K. Liu M.-G. Qiu S. Song Q. O’den G. Chen T.. (2015). Impaired presynaptic long-term potentiation in the anterior cingulate cortex of Fmr1 knock-out mice. J. Neurosci. 35, 2033–2043. 10.1523/JNEUROSCI.2644-14.2015 25653361 PMC6705363

[B141] Kokash J. Alderson E. M. Reinhard S. M. Crawford C. A. Binder D. K. Ethell I. M.. (2019). Genetic reduction of MMP-9 in the Fmr1 KO mouse partially rescues prepulse inhibition of acoustic startle response. Brain Res. 1719, 24–29. 10.1016/j.brainres.2019.05.029 31128097 PMC6640842

[B142] Kozono N. Okamura A. Honda S. Matsumoto M. Mihara T. (2020). Gamma power abnormalities in a Fmr1-targeted transgenic rat model of fragile X syndrome. Sci. Rep. 10:18799. 10.1038/s41598-020-75893-x 33139785 PMC7608556

[B143] Krueger D. D. Bear M. F. (2011). Toward fulfilling the promise of molecular medicine in fragile X syndrome. Annu. Rev. Med. 62, 411–429. 10.1146/annurev-med-061109-134644 21090964 PMC3100156

[B144] Kujirai T. Caramia M. D. Rothwell J. C. Day B. L. Thompson P. D. Ferbert A.. (1993). Corticocortical inhibition in human motor cortex. J. Physiol. 471, 501–519. 10.1113/jphysiol.1993.sp019912 8120818 PMC1143973

[B145] Landau I. D. Egger R. Dercksen V. J. Oberlaender M. Sompolinsky H. (2016). The impact of structural heterogeneity on excitation-inhibition balance in cortical networks. Neuron 92, 1106–1121. 10.1016/j.neuron.2016.10.027 27866797 PMC5158120

[B146] Lauer A. M. Behrens D. Klump G. (2017). Acoustic startle modification as a tool for evaluating auditory function of the mouse: Progress, pitfalls and potential. Neurosci. Biobehav. Rev. 77, 194–208. 10.1016/j.neubiorev.2017.03.009 28327385 PMC5446932

[B147] Lauterborn J. C. Rex C. S. Kramar E. Chen L. Y. Pandyarajan V. Lynch G.. (2007). Brain-derived neurotrophic factor rescues synaptic plasticity in a mouse model of fragile X syndrome. J. Neurosci. 27, 10685–10694. 10.1523/JNEUROSCI.2624-07.2007 17913902 PMC6672822

[B148] Lee H. H. C. Bernard C. Ye Z. Acampora D. Simeone A. Prochiantz A.. (2017). Genetic Otx2 mis-localization delays critical period plasticity across brain regions. Mol. Psychiatry 22:785. 10.1038/mp.2017.83 28373687

[B149] Lee H. Y. Ge W.-P. Huang W. He Y. Wang G. X. Rowson-Baldwin A.. (2011). Bidirectional regulation of dendritic voltage-gated potassium channels by the fragile X mental retardation protein. Neuron 72, 630–642. 10.1016/j.neuron.2011.09.033 22099464 PMC3433402

[B150] Lee K. Y. Jewett K. A. Chung H. J. Tsai N. P. (2018). Loss of fragile X protein FMRP impairs homeostatic synaptic downscaling through tumor suppressor p53 and ubiquitin E3 ligase Nedd4–2. Hum. Mol. Genet. 27, 2805–2816. 10.1093/hmg/ddy189 29771335 PMC6454515

[B151] Leigh M. J. Nguyen D. V. Mu Y. Winarni T. I. Schneider A. Chechi T.. (2013). A randomized double-blind, placebo-controlled trial of minocycline in children and adolescents with fragile x syndrome. J. Dev. Behav. Pediatr. 34, 147–155. 10.1097/DBP.0b013e318287cd17 23572165 PMC3706260

[B152] Lensjo K. K. Lepperod M. E. Dick G. Hafting T. Fyhn M. (2017). Removal of perineuronal nets unlocks juvenile plasticity through network mechanisms of decreased inhibition and increased gamma activity. J. Neurosci. 37, 1269–1283. 10.1523/JNEUROSCI.2504-16.2016 28039374 PMC6596863

[B153] Lin A. Hou Q. Jarzylo L. Amato S. Gilbert J. Shang F.. (2011). Nedd4-mediated AMPA receptor ubiquitination regulates receptor turnover and trafficking. J. Neurochem. 119, 27–39. 10.1111/j.1471-4159.2011.07221.x 21338354 PMC3110981

[B154] Liu D. C. Lee K. Y. Lizarazo S. Cook J. K. Tsai N. P. (2021). ER stress-induced modulation of neural activity and seizure susceptibility is impaired in a fragile X syndrome mouse model. Neurobiol. Dis. 158:105450. 10.1016/j.nbd.2021.105450 34303799 PMC8440443

[B155] Lovelace J. W. Ethell I. M. Binder D. K. Razak K. A. (2018). Translation-relevant EEG phenotypes in a mouse model of Fragile X Syndrome. Neurobiol. Dis. 115, 39–48. 10.1016/j.nbd.2018.03.012 29605426 PMC5969806

[B156] Lovelace J. W. Rais M. Palacios A. R. Shuai X. S. Bishay S. Popa O.. (2020). Deletion of Fmr1 from forebrain excitatory neurons triggers abnormal cellular, eeg and behavioral phenotypes in the auditory cortex of a mouse model of fragile X syndrome. Cereb. Cortex 30, 969–988. 10.1093/cercor/bhz141 31364704 PMC7132927

[B157] Lovelace J. W. Wen T. H. Reinhard S. Hsu M. S. Sidhu H. Ethell I. M.. (2016). Matrix metalloproteinase-9 deletion rescues auditory evoked potential habituation deficit in a mouse model of Fragile X Syndrome. Neurobiol. Dis. 89, 126–135. 10.1016/j.nbd.2016.02.002 26850918 PMC4785038

[B158] Lozano R. Rosero C. A. Hagerman R. J. (2014). Fragile X spectrum disorders. Intractable Rare Dis. Res. 3, 134–146. 10.5582/irdr.2014.01022 25606363 PMC4298643

[B159] Maccarrone M. Rossi S. Bari M. De Chiara V. Rapino C. Musella A.. (2010). Abnormal mGlu 5 receptor/endocannabinoid coupling in mice lacking FMRP and BC1 RNA. Neuropsychopharmacology 35, 1500–1509. 10.1038/npp.2010.19 20393458 PMC3055456

[B160] Maffei A. Nelson S. B. Turrigiano G. G. (2004). Selective reconfiguration of layer 4 visual cortical circuitry by visual deprivation. Nat. Neurosci. 7, 1353–1359. 10.1038/nn1351 15543139

[B161] Marder E. Prinz A. A. (2003). Current compensation in neuronal homeostasis. Neuron 37, 2–4. 10.1016/s0896-6273(02)01173-x 12526765

[B162] Marshall L. Brandt J. F. (1980). The relationship between loudness and reaction time in normal hearing listeners. Acta Otolaryngol. 90, 244–249. 10.3109/00016488009131721 7468185

[B163] Martin B. S. Corbin J. G. Huntsman M. M. (2014). Deficient tonic GABAergic conductance and synaptic balance in the fragile X syndrome amygdala. J. Neurophysiol. 112, 890–902. 10.1152/jn.00597.2013 24848467 PMC4122738

[B164] McCamphill P. K. Stoppel L. J. Senter R. K. Lewis M. C. Heynen A. J. Stoppel D. C.. (2020). Selective inhibition of glycogen synthase kinase 3α corrects pathophysiology in a mouse model of fragile X syndrome. Sci. Transl. Med. 12:eaam8572. 10.1126/scitranslmed.aam8572 32434848 PMC8095719

[B165] Mccullagh E. A. Poleg S. Greene N. T. Huntsman M. M. Tollin D. J. Klug A. (2020). Characterization of auditory and binaural spatial hearing in a fragile X syndrome mouse model. eNeuro 7:ENEURO.0300-0319.2019. 10.1523/ENEURO.0300-19.2019 31953317 PMC7031856

[B166] Mccullagh E. A. Salcedo E. Huntsman M. M. Klug A. (2017). Tonotopic alterations in inhibitory input to the medial nucleus of the trapezoid body in a mouse model of Fragile X syndrome. J. Comp. Neurol. 525, 3543–3562. 10.1002/cne.24290 28744893 PMC5615817

[B167] Mckinney B. C. Grossman A. W. Elisseou N. M. Greenough W. T. (2005). Dendritic spine abnormalities in the occipital cortex of C57BL/6 Fmr1 knockout mice. Am. J. Med. Genet. B. Neuropsychiatr. Genet. 136B, 98–102. 10.1002/ajmg.b.30183 15892134

[B168] Meredith R. M. Holmgren C. D. Weidum M. Burnashev N. Mansvelder H. D. (2007). Increased threshold for spike-timing-dependent plasticity is caused by unreliable calcium signaling in mice lacking fragile X gene Fmr1. Neuron 54, 627–638. 10.1016/j.neuron.2007.04.028 17521574

[B169] Micheva K. D. Beaulieu C. (1996). Quantitative aspects of synaptogenesis in the rat barrel field cortex with special reference to GABA circuitry. J. Comp. Neurol. 373, 340–354. 10.1002/(SICI)1096-9861(19960923)373:3<340::AID-CNE3>3.0.CO;2-2 8889932

[B170] Miller E. A. Kastner D. B. Grzybowski M. N. Dwinell M. R. Geurts A. M. Frank L. M. (2021). Robust and replicable measurement for prepulse inhibition of the acoustic startle response. Mol. Psychiatry 26, 1909–1927. 10.1038/s41380-020-0703-y 32144356 PMC7483293

[B171] Miller L. J. Mcintosh D. N. Mcgrath J. Shyu V. Lampe M. Taylor A. K.. (1999). Electrodermal responses to sensory stimuli in individuals with fragile X syndrome: a preliminary report. Am. J. Med. Genet. 83, 268–279. 10208160

[B172] Morgan P. J. Bourboulou R. Filippi C. Koenig-Gambini J. Epsztein J. (2019). Kv1.1 contributes to a rapid homeostatic plasticity of intrinsic excitability in CA1 pyramidal neurons *in vivo*. eLife 8:e49915. 10.7554/eLife.49915 31774395 PMC6881145

[B173] Morin-Parent F. Champigny C. Lacroix A. Corbin F. Lepage J.-F. (2019). Hyperexcitability and impaired intracortical inhibition in patients with fragile-X syndrome. Transl. Psychiatry 9:312. 10.1038/s41398-019-0650-z 31748507 PMC6868148

[B174] Motanis H. Buonomano D. (2020). Decreased reproducibility and abnormal experience-dependent plasticity of network dynamics in Fragile X circuits. Sci. Rep. 10:14535. 10.1038/s41598-020-71333-y 32884028 PMC7471942

[B175] Murase S. Lantz C. L. Quinlan E. M. (2017). Light reintroduction after dark exposure reactivates plasticity in adults *via* perisynaptic activation of MMP-9. eLife 6:e27345. 10.7554/eLife.27345 28875930 PMC5630258

[B176] Musumeci S. A. Bosco P. Calabrese G. Bakker C. De Sarro G. B. Elia M.. (2000). Audiogenic seizures susceptibility in transgenic mice with fragile X syndrome. Epilepsia 41, 19–23. 10.1111/j.1528-1157.2000.tb01499.x 10643918

[B177] Musumeci S. A. Hagerman R. J. Ferri R. Bosco P. Dalla Bernardina B. Tassinari C. A.. (1999). Epilepsy and EEG findings in males with fragile X syndrome. Epilepsia 40, 1092–1099. 10.1111/j.1528-1157.1999.tb00824.x 10448821

[B178] Myrick L. K. Deng P.-Y. Hashimoto H. Oh Y. M. Cho Y. Poidevin M. J.. (2015). Independent role for presynaptic FMRP revealed by an FMR1 missense mutation associated with intellectual disability and seizures. Proc. Natl. Acad. Sci. U S A 112, 949–956. 10.1073/pnas.1423094112 25561520 PMC4313821

[B179] Myrick L. K. Nakamoto-Kinoshita M. Lindor N. M. Kirmani S. Cheng X. Warren S. T. (2014). Fragile X syndrome due to a missense mutation. Eur. J. Hum. Genet. 22, 1185–1189. 10.1038/ejhg.2013.311 24448548 PMC4169535

[B180] Napoli I. Mercaldo V. Boyl P. P. Eleuteri B. Zalfa F. De Rubeis S.. (2008). The fragile X syndrome protein represses activity-dependent translation through CYFIP1, a new 4E-BP. Cell 134, 1042–1054. 10.1016/j.cell.2008.07.031 18805096

[B181] Nelson S. B. Valakh V. (2015). Excitatory/inhibitory balance and circuit homeostasis in autism spectrum disorders. Neuron 87, 684–698. 10.1016/j.neuron.2015.07.033 26291155 PMC4567857

[B182] Nielsen D. M. Derber W. J. Mcclellan D. A. Crnic L. S. (2002). Alterations in the auditory startle response in Fmr1 targeted mutant mouse models of fragile X syndrome. Brain Res. 927, 8–17. 10.1016/s0006-8993(01)03309-1 11814427

[B183] Niere F. Wilkerson J. R. Huber K. M. (2012). Evidence for a fragile X mental retardation protein-mediated translational switch in metabotropic glutamate receptor-triggered Arc translation and long-term depression. J. Neurosci. 32, 5924–5936. 10.1523/JNEUROSCI.4650-11.2012 22539853 PMC3349238

[B184] Nimchinsky E. A. Oberlander A. M. Svoboda K. (2001). Abnormal development of dendritic spines in FMR1 knock-out mice. J. Neurosci. 21, 5139–5146. 10.1523/JNEUROSCI.21-14-05139.2001 11438589 PMC6762842

[B185] Nosyreva E. D. Huber K. M. (2006). Metabotropic receptor-dependent long-term depression persists in the absence of protein synthesis in the mouse model of fragile X syndrome. J. Neurophysiol. 95, 3291–3295. 10.1152/jn.01316.2005 16452252

[B186] O’donnell C. Goncalves J. T. Portera-Cailliau C. Sejnowski T. J. (2017). Beyond excitation/inhibition imbalance in multidimensional models of neural circuit changes in brain disorders. eLife 6:e26724. 10.7554/eLife.26724 29019321 PMC5663477

[B187] Oh W. C. Smith K. R. (2019). Activity-dependent development of GABAergic synapses. Brain Res. 1707, 18–26. 10.1016/j.brainres.2018.11.014 30439352

[B188] Olmos-Serrano J. L. Corbin J. G. Burns M. P. (2011). The GABA(A) receptor agonist THIP ameliorates specific behavioral deficits in the mouse model of fragile X syndrome. Dev. Neurosci. 33, 395–403. 10.1159/000332884 22067669 PMC3254038

[B189] Olmos-Serrano J. L. Paluszkiewicz S. M. Martin B. S. Kaufmann W. E. Corbin J. G. Huntsman M. M. (2010). Defective GABAergic neurotransmission and pharmacological rescue of neuronal hyperexcitability in the amygdala in a mouse model of fragile X syndrome. J. Neurosci. 30, 9929–9938. 10.1523/JNEUROSCI.1714-10.2010 20660275 PMC2948869

[B190] Orefice L. L. Zimmerman A. L. Chirila A. M. Sleboda S. J. Head J. P. Ginty D. D. (2016). Peripheral mechanosensory neuron dysfunction underlies tactile and behavioral deficits in mouse models of ASDs. Cell 166, 299–313. 10.1016/j.cell.2016.05.033 27293187 PMC5567792

[B191] Osterweil E. K. Chuang S. C. Chubykin A. A. Sidorov M. Bianchi R. Wong R. K.. (2013). Lovastatin corrects excess protein synthesis and prevents epileptogenesis in a mouse model of fragile X syndrome. Neuron 77, 243–250. 10.1016/j.neuron.2012.01.034 23352161 PMC3597444

[B192] Osterweil E. K. Krueger D. D. Reinhold K. Bear M. F. (2010). Hypersensitivity to mGluR5 and ERK1/2 leads to excessive protein synthesis in the hippocampus of a mouse model of fragile X syndrome. J. Neurosci. 30, 15616–15627. 10.1523/JNEUROSCI.3888-10.2010 21084617 PMC3400430

[B193] Pak A. Kissinger S. T. Chubykin A. A. (2021). Impaired adaptation and laminar processing of the oddball paradigm in the primary visual cortex of Fmr1 KO mouse. Front. Cell. Neurosci. 15:668230. 10.3389/fncel.2021.668230 34093132 PMC8170411

[B194] Paluszkiewicz S. M. Olmos-Serrano J. L. Corbin J. G. Huntsman M. M. (2011). Impaired inhibitory control of cortical synchronization in fragile X syndrome. J. Neurophysiol. 106, 2264–2272. 10.1152/jn.00421.2011 21795626 PMC3214096

[B195] Pan F. Aldridge G. M. Greenough W. T. Gan W.-B. (2010). Dendritic spine instability and insensitivity to modulation by sensory experience in a mouse model of fragile X syndrome. Proc. Natl. Acad. Sci. U S A 107, 17768–17773. 10.1073/pnas.1012496107 20861447 PMC2955121

[B196] Pan L. Zhang Y. Q. Woodruff E. Broadie K. (2004). The drosophila fragile X gene negatively regulates neuronal elaboration and synaptic differentiation. Curr. Biol. 14, 1863–1870. 10.1016/j.cub.2004.09.085 15498496

[B197] Paradee W. Melikian H. E. Rasmussen D. L. Kenneson A. Conn P. J. Warren S. T. (1999). Fragile X mouse: strain effects of knockout phenotype and evidence suggesting deficient amygdala function. Neuroscience 94, 185–192. 10.1016/s0306-4522(99)00285-7 10613508

[B199] Park T. J. Grothe B. Pollak G. D. Schuller G. Koch U. (1996). Neural delays shape selectivity to interaural intensity differences in the lateral superior olive. J. Neurosci. 16, 6554–6566. 10.1523/JNEUROSCI.16-20-06554.1996 8815932 PMC6578907

[B198] Park E. Lau A. G. Arendt K. L. Chen L. (2021). Fmrp interacts with rarα in synaptic retinoic acid signaling and homeostatic synaptic plasticity. Int. J. Mol. Sci. 22:6579. 10.1038/s41386-018-0150-5 34205274 PMC8235556

[B200] Partanen E. Kujala T. Naatanen R. Liitola A. Sambeth A. Huotilainen M. (2013). Learning-induced neural plasticity of speech processing before birth. Proc. Natl. Acad. Sci. U S A 110, 15145–15150. 10.1073/pnas.1302159110 23980148 PMC3773755

[B201] Patel A. B. Hays S. A. Bureau I. Huber K. M. Gibson J. R. (2013). A target cell-specific role for presynaptic Fmr1 in regulating glutamate release onto neocortical fast-spiking inhibitory neurons. J. Neurosci. 33, 2593–2604. 10.1523/JNEUROSCI.2447-12.2013 23392687 PMC3711607

[B202] Patel A. B. Loerwald K. W. Huber K. M. Gibson J. R. (2014). Postsynaptic FMRP promotes the pruning of cell-to-cell connections among pyramidal neurons in the L5A neocortical network. J. Neurosci. 34, 3413–3418. 10.1523/JNEUROSCI.2921-13.2014 24573297 PMC3935093

[B203] Paylor R. Yuva-Paylor L. A. Nelson D. L. Spencer C. M. (2008). Reversal of sensorimotor gating abnormalities in Fmr1 knockout mice carrying a human Fmr1 transgene. Behav. Neurosci. 122, 1371–1377. 10.1037/a0013047 19045956

[B204] Pfeffer C. K. Xue M. He M. Huang Z. J. Scanziani M. (2013). Inhibition of inhibition in visual cortex: the logic of connections between molecularly distinct interneurons. Nat. Neurosci. 16, 1068–1076. 10.1038/nn.3446 23817549 PMC3729586

[B205] Pfeiffer B. E. Zang T. Wilkerson J. R. Taniguchi M. Maksimova M. A. Smith L. N.. (2010). Fragile X mental retardation protein is required for synapse elimination by the activity-dependent transcription factor MEF2. Neuron 66, 191–197. 10.1016/j.neuron.2010.03.017 20434996 PMC2864778

[B206] Pinard A. Seddik R. Bettler B. (2010). GABAB receptors: physiological functions and mechanisms of diversity. Adv. Pharmacol. 58, 231–255. 10.1016/S1054-3589(10)58010-4 20655485

[B207] Pizzorusso T. Medini P. Berardi N. Chierzi S. Fawcett J. W. Maffei L. (2002). Reactivation of ocular dominance plasticity in the adult visual cortex. Science 298, 1248–1251. 10.1126/science.1072699 12424383

[B209] Qiu L. F. Hao Y. H. Li Q. Z. Xiong Z. Q. (2008). Fragile X syndrome and epilepsy. Neurosci. Bull. 24, 338–344. 10.1007/s12264-008-1221-0 18839028 PMC5552528

[B210] Qiu L. F. Lu T. J. Hu X. L. Yi Y. H. Liao W. P. Xiong Z. Q. (2009). Limbic epileptogenesis in a mouse model of fragile X syndrome. Cereb. Cortex 19, 1504–1514. 10.1093/cercor/bhn163 18832330 PMC2693616

[B208] Qin M. Schmidt K. C. Zametkin A. J. Bishu S. Horowitz L. M. Burlin T. V.. (2013). Altered cerebral protein synthesis in fragile X syndrome: studies in human subjects and knockout mice. J. Cereb. Blood Flow Metab. 33, 499–507. 10.1038/jcbfm.2012.205 23299245 PMC3618394

[B211] Radziwon K. Salvi R. (2020). Using auditory reaction time to measure loudness growth in rats. Hear. Res. 395:108026. 10.1016/j.heares.2020.108026 32668383

[B212] Rais M. Binder D. K. Razak K. A. Ethell I. M. (2018). Sensory processing phenotypes in fragile X syndrome. ASN Neuro 10:1759091418801092. 10.1177/1759091418801092 30231625 PMC6149018

[B213] Raymond C. R. Thompson V. L. Tate W. P. Abraham W. C. (2000). Metabotropic glutamate receptors trigger homosynaptic protein synthesis to prolong long-term potentiation. J. Neurosci. 20, 969–976. 10.1523/JNEUROSCI.20-03-00969.2000 10648701 PMC6774154

[B214] Represa A. Ben-Ari Y. (2005). Trophic actions of GABA on neuronal development. Trends Neurosci. 28, 278–283. 10.1016/j.tins.2005.03.010 15927682

[B215] Rio C. A. C.-D. Nunez-Parra A. Freedman S. Restrepo D. Huntsman M. M. (2018). Homeostatic inhibitory control of cortical hyperexcitability in fragile X syndrome. bioRxiv [Preprint]. 10.1101/459511

[B216] Rodriguez G. Mesik L. Gao M. Parkins S. Saha R. Lee H. K. (2019). Disruption of NMDAR function prevents normal experience-dependent homeostatic synaptic plasticity in mouse primary visual cortex. J. Neurosci. 39, 7664–7673. 10.1523/JNEUROSCI.2117-18.2019 31413075 PMC6764196

[B217] Rogers S. J. Hepburn S. Wehner E. (2003). Parent reports of sensory symptoms in toddlers with autism and those with other developmental disorders. J. Autism Dev. Disord. 33, 631–642. 10.1023/b:jadd.0000006000.38991.a7 14714932

[B218] Rotschafer S. Razak K. (2013). Altered auditory processing in a mouse model of fragile X syndrome. Brain Res. 1506, 12–24. 10.1016/j.brainres.2013.02.038 23458504

[B219] Routh B. N. Johnston D. Brager D. H. (2013). Loss of functional A-type potassium channels in the dendrites of CA1 pyramidal neurons from a mouse model of fragile X syndrome. J. Neurosci. 33, 19442–19450. 10.1523/JNEUROSCI.3256-13.2013 24336711 PMC3858620

[B220] Routh B. N. Rathour R. K. Baumgardner M. E. Kalmbach B. E. Johnston D. Brager D. H. (2017). Increased transient Na+ conductance and action potential output in layer 2/3 prefrontal cortex neurons of the fmr1-/y mouse. J. Physiol. 595, 4431–4448. 10.1113/JP274258 28370141 PMC5491866

[B221] Rutherford L. C. Nelson S. B. Turrigiano G. G. (1998). BDNF has opposite effects on the quantal amplitude of pyramidal neuron and interneuron excitatory synapses. Neuron 21, 521–530. 10.1016/s0896-6273(00)80563-2 9768839

[B222] Saffary R. Xie Z. (2011). FMRP regulates the transition from radial glial cells to intermediate progenitor cells during neocortical development. J. Neurosci. 31, 1427–1439. 10.1523/JNEUROSCI.4854-10.2011 21273427 PMC6623593

[B223] Sakai J. (2020). Core concept: how synaptic pruning shapes neural wiring during development and, possibly, in disease. Proc. Nat. Acad. Sci. U S A 117, 16096–16099. 10.1073/pnas.2010281117 32581125 PMC7368197

[B224] Salkoff L. Butler A. Ferreira G. Santi C. Wei A. (2006). High-conductance potassium channels of the SLO family. Nat. Rev. Neurosci. 7, 921–931. 10.1038/nrn1992 17115074

[B225] Santoro M. R. Bray S. M. Warren S. T. (2012). Molecular mechanisms of fragile X syndrome: a twenty-year perspective. Annu. Rev. Pathol. 7, 219–245. 10.1146/annurev-pathol-011811-132457 22017584

[B226] Schilit Nitenson A. Stackpole E. E. Truszkowski T. L. S. Midroit M. Fallon J. R. Bath K. G. (2015). Fragile X mental retardation protein regulates olfactory sensitivity but not odorant discrimination. Chem. Senses 40, 345–350. 10.1093/chemse/bjv019 25917509 PMC4542900

[B227] Schroeder J. C. Reim D. Boeckers T. M. Schmeisser M. J. (2017). Genetic animal models for autism spectrum disorder. Curr. Top. Behav. Neurosci. 30, 311–324. 10.1007/7854_2015_407 26602248

[B228] Schwarz L. A. Hall B. J. Patrick G. N. (2010). Activity-dependent ubiquitination of GluA1 mediates a distinct AMPA receptor endocytosis and sorting pathway. J. Neurosci. 30, 16718–16729. 10.1523/JNEUROSCI.3686-10.2010 21148011 PMC3079366

[B229] Selby L. Zhang C. Sun Q.-Q. (2007). Major defects in neocortical GABAergic inhibitory circuits in mice lacking the fragile X mental retardation protein. Neurosci. Lett. 412, 227–232. 10.1016/j.neulet.2006.11.062 17197085 PMC1839948

[B230] Shah M. M. (2014). Cortical HCN channels: function, trafficking and plasticity. J. Physiol. 592, 2711–2719. 10.1113/jphysiol.2013.270058 24756635 PMC4104471

[B231] Shang Y. Wang H. Mercaldo V. Li X. Chen T. Zhuo M. (2009). Fragile X mental retardation protein is required for chemically-induced long-term potentiation of the hippocampus in adult mice. J. Neurochem. 111, 635–646. 10.1111/j.1471-4159.2009.06314.x 19659572

[B232] Shepherd J. D. Rumbaugh G. Wu J. Chowdhury S. Plath N. Kuhl D.. (2006). Arc/Arg3.1 mediates homeostatic synaptic scaling of AMPA receptors. Neuron 52, 475–484. 10.1016/j.neuron.2006.08.034 17088213 PMC1764219

[B233] Shew W. L. Yang H. Yu S. Roy R. Plenz D. (2011). Information capacity and transmission are maximized in balanced cortical networks with neuronal avalanches. J. Neurosci. 31, 55–63. 10.1523/JNEUROSCI.4637-10.2011 21209189 PMC3082868

[B234] Sidorov M. S. Auerbach B. D. Bear M. F. (2013). Fragile X mental retardation protein and synaptic plasticity. Mol. Brain 6:15. 10.1186/1756-6606-6-15 23566911 PMC3636002

[B235] Siller S. S. Broadie K. (2011). Neural circuit architecture defects in a Drosophila model of Fragile X syndrome are alleviated by minocycline treatment and genetic removal of matrix metalloproteinase. Dis. Model. Mech. 4, 673–685. 10.1242/dmm.008045 21669931 PMC3180232

[B236] Silverman J. L. Pride M. C. Hayes J. E. Puhger K. R. Butler-Struben H. M. Baker S.. (2015). GABAB receptor agonist R-baclofen reverses social deficits and reduces repetitive behavior in two mouse models of autism. Neuropsychopharmacology 40, 2228–2239. 10.1038/npp.2015.66 25754761 PMC4613612

[B237] Sinclair D. Featherstone R. Naschek M. Nam J. Du A. Wright S.. (2017a). GABA-B agonist baclofen normalizes auditory-evoked neural oscillations and behavioral deficits in the Fmr1 knockout mouse model of fragile X syndrome. eNeuro 4:ENEURO.0380-16.2017. 10.1523/ENEURO.0380-16.2017 28451631 PMC5394929

[B238] Sinclair D. Oranje B. Razak K. A. Siegel S. J. Schmid S. (2017b). Sensory processing in autism spectrum disorders and fragile X syndrome-from the clinic to animal models. Neurosci. Biobehav. Rev. 76, 235–253. 10.1016/j.neubiorev.2016.05.029 27235081 PMC5465967

[B239] Siomi H. Siomi M. C. Nussbaum R. L. Dreyfuss G. (1993). The protein product of the fragile X gene, FMR1, has characteristics of an RNA-binding protein. Cell 74, 291–298. 10.1016/0092-8674(93)90420-u 7688265

[B240] Snyder E. M. Philpot B. D. Huber K. M. Dong X. Fallon J. R. Bear M. F. (2001). Internalization of ionotropic glutamate receptors in response to mGluR activation. Nat. Neurosci. 4, 1079–1085. 10.1038/nn746 11687813

[B241] Soden M. E. Chen L. (2010). Fragile X protein FMRP is required for homeostatic plasticity and regulation of synaptic strength by retinoic acid. J. Neurosci. 30, 16910–16921. 10.1523/JNEUROSCI.3660-10.2010 21159962 PMC3073636

[B242] Sohal V. S. Zhang F. Yizhar O. Deisseroth K. (2009). Parvalbumin neurons and gamma rhythms enhance cortical circuit performance. Nature 459, 698–702. 10.1038/nature07991 19396159 PMC3969859

[B243] Stefani G. Fraser C. E. Darnell J. C. Darnell R. B. (2004). Fragile X mental retardation protein is associated with translating polyribosomes in neuronal cells. J. Neurosci. 24, 7272–7276. 10.1523/JNEUROSCI.2306-04.2004 15317853 PMC6729764

[B244] Stellwagen D. Malenka R. C. (2006). Synaptic scaling mediated by glial TNF-α. Nature 440, 1054–1059. 10.1038/nature04671 16547515

[B245] Stoppel D. C. Mccamphill P. K. Senter R. K. Heynen A. J. Bear M. F. (2021). mGluR5 negative modulators for fragile X: treatment resistance and persistence. Front. Psychiatry 12:718953. 10.3389/fpsyt.2021.718953 34658956 PMC8511445

[B246] Strumbos J. G. Brown M. R. Kronengold J. Polley D. B. Kaczmarek L. K. (2010). Fragile X mental retardation protein is required for rapid experience-dependent regulation of the potassium channel Kv3.1b. J. Neurosci. 30, 10263–10271. 10.1523/JNEUROSCI.1125-10.2010 20685971 PMC3485078

[B247] Stuart G. J. Spruston N. (2015). Dendritic integration: 60 years of progress. Nat. Neurosci. 18, 1713–1721. 10.1038/nn.4157 26605882

[B248] Suhl J. A. Warren S. T. (2015). Single-nucleotide mutations in FMR1 reveal novel functions and regulatory mechanisms of the fragile X syndrome protein FMRP. J. Exp. Neurosci. 9, 35–41. 10.4137/JEN.S25524 26819560 PMC4720182

[B249] Sun Q. Turrigiano G. G. (2011). PSD-95 and PSD-93 play critical but distinct roles in synaptic scaling up and down. J. Neurosci. 31, 6800–6808. 10.1523/JNEUROSCI.5616-10.2011 21543610 PMC3113607

[B250] Svalina M. N. Guthman E. M. Cea-Del Rio C. A. Kushner J. K. Baca S. M. Restrepo D.. (2021). Hyperexcitability and loss of feedforward inhibition contribute to aberrant plasticity in the Fmr1KO amygdala. eNeuro 8:ENEURO.0113–0121.2021. 10.1523/ENEURO.0113-21.2021 33893168 PMC8121259

[B251] Telias M. Segal M. Ben-Yosef D. (2013). Neural differentiation of Fragile X human embryonic stem cells reveals abnormal patterns of development despite successful neurogenesis. Dev. Biol. 374, 32–45. 10.1016/j.ydbio.2012.11.031 23219959

[B252] Till S. M. (2010). The developmental roles of FMRP. Biochem. Soc. Trans. 38, 507–510. 10.1042/BST0380507 20298211

[B253] Till S. M. Asiminas A. Jackson A. D. Katsanevaki D. Barnes S. A. Osterweil E. K.. (2015). Conserved hippocampal cellular pathophysiology but distinct behavioural deficits in a new rat model of FXS. Hum. Mol. Genet. 24, 5977–5984. 10.1093/hmg/ddv299 26243794 PMC4599667

[B254] Todd P. K. Mack K. J. Malter J. S. (2003). The fragile X mental retardation protein is required for type-I metabotropic glutamate receptor-dependent translation of PSD-95. Proc. Natl. Acad. Sci. U S A 100, 14374–14378. 10.1073/pnas.2336265100 14614133 PMC283599

[B255] Tsai N. P. Wilkerson J. R. Guo W. Huber K. M. (2017). FMRP-dependent Mdm2 dephosphorylation is required for MEF2-induced synapse elimination. Hum. Mol. Genet. 26, 293–304. 10.1093/hmg/ddw386 28025327 PMC6075181

[B256] Tsai N. P. Wilkerson J. R. Guo W. Maksimova M. A. Demartino G. N. Cowan C. W.. (2012). Multiple autism-linked genes mediate synapse elimination *via* proteasomal degradation of a synaptic scaffold PSD-95. Cell 151, 1581–1594. 10.1016/j.cell.2012.11.040 23260144 PMC3530171

[B257] Turrigiano G. G. (2008). The self-tuning neuron: synaptic scaling of excitatory synapses. Cell 135, 422–435. 10.1016/j.cell.2008.10.008 18984155 PMC2834419

[B258] Turrigiano G. G. Leslie K. R. Desai N. S. Rutherford L. C. Nelson S. B. (1998). Activity-dependent scaling of quantal amplitude in neocortical neurons. Nature 391, 892–896. 10.1038/36103 9495341

[B259] Turrigiano G. G. Nelson S. B. (2000). Hebb and homeostasis in neuronal plasticity. Curr. Opin. Neurobiol. 10, 358–364. 10.1016/s0959-4388(00)00091-x 10851171

[B260] Turrigiano G. G. Nelson S. B. (2004). Homeostatic plasticity in the developing nervous system. Nat. Rev. Neurosci. 5, 97–107. 10.1038/nrn1327 14735113

[B261] Typlt M. Mirkowski M. Azzopardi E. Ruth P. Pilz P. K. Schmid S. (2013). Habituation of reflexive and motivated behavior in mice with deficient BK channel function. Front. Integr. Neurosci. 7:79. 10.3389/fnint.2013.00079 24312024 PMC3833254

[B262] Tyzio R. Nardou R. Ferrari D. C. Tsintsadze T. Shahrokhi A. Eftekhari S.. (2014). Oxytocin-mediated GABA inhibition during delivery attenuates autism pathogenesis in rodent offspring. Science 343, 675–679. 10.1126/science.1247190 24503856

[B263] Van der Molen M. J. Van der Molen M. W. Ridderinkhof K. R. Hamel B. C. Curfs L. M. Ramakers G. J. (2012). Auditory change detection in fragile X syndrome males: a brain potential study. Clin. Neurophysiol. 123, 1309–1318. 10.1016/j.clinph.2011.11.039 22192499

[B264] Verheij C. De Graaff E. Bakker C. E. Willemsen R. Willems P. J. Meijer N.. (1995). Characterization of FMR1 proteins isolated from different tissues. Hum. Mol. Genet. 4, 895–901. 10.1093/hmg/4.5.895 7633450

[B265] Vislay R. L. Martin B. S. Olmos-Serrano J. L. Kratovac S. Nelson D. L. Corbin J. G.. (2013). Homeostatic responses fail to correct defective amygdala inhibitory circuit maturation in fragile X syndrome. J. Neurosci. 33, 7548–7558. 10.1523/JNEUROSCI.2764-12.2013 23616559 PMC3684185

[B266] Vitureira N. Goda Y. (2013). Cell biology in neuroscience: The interplay between Hebbian and homeostatic synaptic plasticity. J. Cell Biol. 203, 175–186. 10.1083/jcb.201306030 24165934 PMC3812972

[B267] Wahlstrom-Helgren S. Klyachko V. A. (2015). GABAB receptor-mediated feed-forward circuit dysfunction in the mouse model of fragile X syndrome. J. Physiol. 593, 5009–5024. 10.1113/JP271190 26282581 PMC4650406

[B268] Wahlstrom-Helgren S. Klyachko V. A. (2016). Dynamic balance of excitation and inhibition rapidly modulates spike probability and precision in feed-forward hippocampal circuits. J. Neurophysiol. 116, 2564–2575. 10.1152/jn.00413.2016 27605532 PMC5133295

[B269] Wang J. Ethridge L. E. Mosconi M. W. White S. P. Binder D. K. Pedapati E. V.. (2017). A resting EEG study of neocortical hyperexcitability and altered functional connectivity in fragile X syndrome. J. Neurodev. Disord. 9:11. 10.1186/s11689-017-9191-z 28316753 PMC5351111

[B270] Wang X. Zorio D. A. R. Schecterson L. Lu Y. Wang Y. (2018). Postsynaptic FMRP regulates synaptogenesis *in vivo* in the developing cochlear nucleus. J. Neurosci. 38, 6445–6460. 10.1523/JNEUROSCI.0665-18.2018 29950504 PMC6052239

[B271] Watt A. J. Van Rossum M. C. W. Macleod K. M. Nelson S. B. Turrigiano G. G. (2000). Activity coregulates quantal AMPA and NMDA currents at neocortical synapses. Neuron 26, 659–670. 10.1016/s0896-6273(00)81202-7 10896161

[B272] Weiler I. J. Irwin S. A. Klintsova A. Y. Spencer C. M. Brazelton A. D. Miyashiro K.. (1997). Fragile X mental retardation protein is translated near synapses in response to neurotransmitter activation. Proc. Natl. Acad. Sci. U S A 94, 5395–5400. 10.1073/pnas.94.10.5395 9144248 PMC24689

[B273] Wen T. H. Afroz S. Reinhard S. M. Palacios A. R. Tapia K. Binder D. K.. (2018). Genetic reduction of matrix metalloproteinase-9 promotes formation of perineuronal nets around parvalbumin-expressing interneurons and normalizes auditory cortex responses in developing Fmr1 knock-out mice. Cereb. Cortex 28, 3951–3964. 10.1093/cercor/bhx258 29040407 PMC6188540

[B274] Wierenga C. J. Ibata K. Turrigiano G. G. (2005). Postsynaptic expression of homeostatic plasticity at neocortical synapses. J. Neurosci. 25, 2895–2905. 10.1523/JNEUROSCI.5217-04.2005 15772349 PMC6725152

[B275] Wisniewski K. E. Segan S. M. Miezejeski C. M. Sersen E. A. Rudelli R. D. (1991). The Fra(X) syndrome: neurological, electrophysiological and neuropathological abnormalities. Am. J. Med. Genet. 38, 476–480. 10.1002/ajmg.1320380267 2018089

[B276] Wong H. Hooper A. W. M. Niibori Y. Lee S. J. Hategan L. A. Zhang L.. (2020). Sexually dimorphic patterns in electroencephalography power spectrum and autism-related behaviors in a rat model of fragile X syndrome. Neurobiol. Dis. 146:105118. 10.1016/j.nbd.2020.105118 33031903

[B2881] Yang Y.-M. Arsenault J. Bah A. Krzeminski M. Fekete A. Chao O. Y.. (2020). Identification of a molecular locus for normalizing dysregulated GABA release from interneurons in the Fragile X brain. Mol. Psychiatry 25, 2017–2035. 10.1038/s41380-018-0240-0 30224722 PMC7473840

[B277] Yang S. Yang S. Park J. S. Kirkwood A. Bao S. (2014). Failed stabilization for long-term potentiation in the auditory cortex of FMR1 knockout mice. PLoS One 9:e104691. 10.1371/journal.pone.0104691 25115962 PMC4130563

[B278] Yizhar O. Fenno L. E. Prigge M. Schneider F. Davidson T. J. O’shea D. J.. (2011). Neocortical excitation/inhibition balance in information processing and social dysfunction. Nature 477, 171–178. 10.1038/nature10360 21796121 PMC4155501

[B279] Yun S. W. Platholi J. Flaherty M. S. Fu W. Kottmann A. H. Toth M. (2006). Fmrp is required for the establishment of the startle response during the critical period of auditory development. Brain Res. 1110, 159–165. 10.1016/j.brainres.2006.06.086 16887106

[B2850] Zeidler S. Pop A. S. Jaafar I. A. de Boer H. Buijsen R. A. M. de Esch C. E. F.. (2018). Paradoxical effect of baclofen on social behavior in the fragile X syndrome mouse model. Brain Behav. 8:e00991. 10.1002/brb3.991 29785777 PMC5991574

[B280] Zeier Z. Kumar A. Bodhinathan K. Feller J. A. Foster T. C. Bloom D. C. (2009). Fragile X mental retardation protein replacement restores hippocampal synaptic function in a mouse model of fragile X syndrome. Gene. Ther. 16, 1122–1129. 10.1038/gt.2009.83 19571888 PMC2741536

[B281] Zhan X. Asmara H. Cheng N. Sahu G. Sanchez E. Zhang F.-X.. (2020). FMRP(1–297)-tat restores ion channel and synaptic function in a model of Fragile X syndrome. Nat. Commun. 11:2755. 10.1038/s41467-020-16250-4 32488011 PMC7265297

[B282] Zhang L. Alger B. E. (2010). Enhanced endocannabinoid signaling elevates neuronal excitability in fragile X syndrome. J. Neurosci. 30, 5724–5729. 10.1523/JNEUROSCI.0795-10.2010 20410124 PMC2906112

[B286] Zhang Y. Q. Bailey A. M. Matthies H. J. G. Renden R. B. Smith M. A. Speese S. D.. (2001). Drosophila fragile X-related gene regulates the MAP1B homolog futsch to control synaptic structure and function. Cell 107, 591–603. 10.1016/s0092-8674(01)00589-x 11733059

[B283] Zhang L. I. Bao S. Merzenich M. M. (2001). Persistent and specific influences of early acoustic environments on primary auditory cortex. Nat. Neurosci. 4, 1123–1130. 10.1038/nn745 11687817

[B285] Zhang Y. Bonnan A. Bony G. Ferezou I. Pietropaolo S. Ginger M.. (2014). Dendritic channelopathies contribute to neocortical and sensory hyperexcitability in Fmr1(-/y) mice. Nat. Neurosci. 17, 1701–1709. 10.1038/nn.3864 25383903

[B284] Zhang W. Linden D. J. (2003). The other side of the engram: experience-driven changes in neuronal intrinsic excitability. Nat. Rev. Neurosci. 4, 885–900. 10.1038/nrn1248 14595400

[B287] Zhang Z. Marro S. G. Zhang Y. Arendt K. L. Patzke C. Zhou B.. (2018). The fragile X mutation impairs homeostatic plasticity in human neurons by blocking synaptic retinoic acid signaling. Sci. Trans. Med. 10:eaar4338. 10.1126/scitranslmed.aar4338 30068571 PMC6317709

[B288] Zhang Z. Rumschlag J. Jonak C. R. Binder D. K. Razak K. A. Gibson J. R.. (2021). FMRP regulates experience-dependent maturation of callosal synaptic connections and bilateral cortical synchrony. BioRxiv [Preprint]. 10.1101/2021.06.25.449490

[B289] Zhao W. Chuang S.-C. Bianchi R. Wong R. K. S. (2011). Dual regulation of fragile X mental retardation protein by group I metabotropic glutamate receptors controls translation-dependent epileptogenesis in the hippocampus. J. Neurosci. 31, 725–734. 10.1523/JNEUROSCI.2915-10.2011 21228181 PMC6623451

[B290] Zhong L. R. Chen X. Park E. Südhof T. C. Chen L. (2018). Retinoic acid receptor RARα-dependent synaptic signaling mediates homeostatic synaptic plasticity at the inhibitory synapses of mouse visual cortex. J. Neurosci. 38, 10454–10466. 10.1523/JNEUROSCI.1133-18.2018 30355624 PMC6284108

